# Energy-Efficient Hybrid Precoding with Switches and Inverters for Millimeter-Wave MIMO Systems

**DOI:** 10.3390/s26216895

**Published:** 2026-10-30

**Authors:** Sining Li, Ke Xu, Huilin Ren, Changwei Huang, Dan Wu, Zhuoqiao Ji, Richu Jin, Bochao Su, Changning Wei, Weiqiang Wu

**Affiliations:** 1 Shenzhen Institutes of Advanced Technology, Chinese Academy of Sciences, Shenzhen 518055, Chinarenhuilin@szpu.edu.cn (H.R.); dan.wu@siat.ac.cn (D.W.); 2 Tech X Academy, Shenzhen Polytechnic University, Shenzhen 518055, China; huangcw@szpu.edu.cn (C.H.); jinrichu@szpu.edu.cn (R.J.); subochao@szpu.edu.cn (B.S.); 3 School of Electronic and Communication Engineering, Shenzhen Polytechnic University, Shenzhen 518055, Chinawuweiqiang@szpu.edu.cn (W.W.); 4 School of Mechanical and Electrical Engineering, Shenzhen Polytechnic University, Shenzhen 518055, China; jizhuoqiao@szpu.edu.cn

**Keywords:** dynamic programming, energy efficiency, hybrid precoding, massive MIMO, millimeter-wave MIMO, switches and inverters

## Abstract

This paper investigates low-complexity hybrid precoding for a millimeter-wave multi-user massive multiple-input-multiple-output (MIMO) downlink, where a base station simultaneously serves multiple single-antenna users over frequency-flat narrowband channels. The design seeks to improve the trade-off between achievable sum rate, power consumption, and computational cost using a switch-inverter (SI) architecture that replaces high-resolution phase shifters (PSs) with switches and inverters. Digital zero-forcing (ZF) precoding addresses inter-user interference through the effective channel, while a dynamic programming (DP)-based sequential procedure selects SI states for local analog beamforming. The procedure reuses partial complex sums and performs exact amplitude comparisons within a restricted candidate set, without a guarantee of global optimality. Energy efficiency (EE) is evaluated as the bandwidth-normalized achievable sum rate divided by the total consumed power. To improve this metric, the revised DP procedure is combined with antenna deactivation under prescribed analog beamforming-gain constraints. This gain-based design does not directly maximize the multi-user sum rate or EE. For the evaluated 128-antenna SI array, antenna deactivation yields an observed EE improvement of approximately 15% relative to the corresponding all-active DP configuration. Coupling between active and inactive antenna elements is considered, and a corresponding feeding-voltage adjustment is derived to maintain the beam pattern. Applicability to wideband transmission is discussed through a conditional analysis of local analog beamforming gain.

## 1. Introduction

The use of massive antenna arrays in millimeter-wave (mmWave) multiple-input-multiple-output (MIMO) systems helps compensate for the relatively high path loss in mmWave frequency bands [1,2,3].

Throughout this paper, precoding denotes the linear mapping of user data streams to antenna signals, while beamforming emphasizes the spatial weighting used to shape the radiation pattern and obtain directional gain. These terms describe closely related aspects of spatial processing, and beamforming can be implemented through analog or digital precoding [3,4].

In a hybrid transmitter, the digital baseband precoder first maps the user streams to the RF-chain signals using complex-valued weights. Digital-to-analog converters (DACs) and RF upconversion then produce the signals supplied to the analog network, whose weights are constrained by the phase shifters, switches, or inverters used in that network. The stages can be cascaded because the digital precoder has $N_{RF}$ outputs and the analog precoder has $N_{RF}$ inputs; their product is the overall stream-to-antenna mapping. Hybrid processing can, therefore, retain digital stream coordination while connecting a large array through fewer RF chains than antennas. The performance depends on the available RF chains and analog constraints, rather than being universally equivalent to unrestricted fully digital processing [5,6].

In single-user MIMO, the design concerns the spatial streams intended for one receiver; in multi-user downlink transmission, it also coordinates streams intended for different users and suppresses inter-user interference. This work considers a base station (BS) simultaneously serving multiple single-antenna users with one stream per user. Digital zero-forcing (ZF) precoding is applied to the effective channel formed after analog processing; with perfect channel state information (CSI) and a full-row-rank effective channel, it cancels inter-user interference. The proposed dynamic programming (DP) procedure selects switch-inverter (SI) states within fixed subarrays using local analog beamforming-gain comparisons. The analog and digital stages jointly determine the effective antenna weights for each user. The design seeks useful sum-rate performance with low computational and circuit-power costs, and uses antenna deactivation under prescribed analog-gain constraints to pursue energy efficiency (EE) improvements. Here, EE always denotes the achievable sum rate per unit bandwidth divided by the total consumed power. Active-antenna count provides a circuit-power proxy for the antenna-deactivation design, while EE evaluates the resulting rate–power trade-off. These local analog objectives do not establish global maximization of the multi-user sum rate or EE.

Switch-only analog paths use binary coefficients in {0,1}: 0 denotes a disconnected path and 1 a path carrying the original signal [7]. Adding an inverter permits the coefficient −1, so controllable SI paths take values in {−1,0,1} for inverted, inactive, and non-inverted states, respectively. The resulting discrete hardware decisions motivate a computationally economical analog-state design. Section 2 reviews the associated architectures and algorithms and compares their design variables with those considered here.

For the evaluated $N_{BS}=128$, $K=N_{RF}=4$ all-active setting at SNR = 20 dB, the existing curves give approximate sum-rate gains of 9.9% and 10.0% over CU in the urban and rural cases, and 20.6% and 17.4% over CE, respectively. Antenna deactivation gives an observed EE improvement of approximately 15% relative to the corresponding all-active DP configuration in the settings, at SNR = 10 dB. For one local all-active subarray pass, sorting costs $O(n_{i}\log n_{i})$ on average and sequential comparison costs $O(n_{i})$; at most $4(n_{i}-1)=124$ local amplitude evaluations are required for $n_{i}=32$. Processing each of the four subarrays once gives at most 496 local evaluations for the complete array, or 3.875 per installed antenna. Deactivation updates, feasibility checks, and digital processing incur additional costs. These are local workload measures, not execution-time ratios against complete system-objective evaluations.

Potential applications include cost- and power-conscious mmWave access points and sensor-network gateways serving multiple downlink control or data streams. Industrial connectivity motivates attention to beam management and propagation conditions [8]. The present scheme addresses transmitter-side analog-state and activation decisions within the frequency-flat model and available-CSI assumptions; its application requires those assumptions to remain valid over the design and transmission interval. General wideband operation needs the joint analog–digital treatment discussed in Section 4.5. Major contributions of this work can be summarized as follows:

- We propose a low-complexity DP-based sequential precoding scheme for the switch-inverter array architecture. The scheme reuses partial complex sums to select antenna states recursively. Antenna ordering and additional candidate comparisons are introduced to mitigate performance loss caused by discarding intermediate states. We analyze the mechanism of suboptimality and evaluate the resulting performance and computational complexity.
- We develop a QoS-constrained antenna-deactivation strategy integrated with the revised DP precoder. The strategy uses beamforming-contribution estimates obtained from the DP states to guide sequential antenna deactivation subject to a prescribed beamforming-gain constraint. Under the investigated channel and power-consumption settings, we analyze how the QoS threshold, propagation-path structure, and RF-chain power consumption affect antenna deactivation and the achievable rate–EE trade-off. The resulting observations provide practical guidance on selecting a deactivation strategy and setting the QoS threshold.

The rest of this paper is organized as follows. Section 2 reviews previous related work and positions the proposed method. Section 3 presents the system model. Section 4 and Section 5 develop the all-active SI analog design and the QoS-constrained antenna-deactivation scheme, respectively, including their analytical limitations and computational workload. Section 6 reports the numerical results and design insights. Section 7 concludes the paper.

*Notations*: Bold uppercase symbols such as A denote matrices, bold lowercase symbols such as a denote vectors, and ordinary mathematical italic symbols denote scalars. Calligraphic symbols such as A denote sets. Superscripts *H*, *T*, and ∗ denote conjugate transpose, transpose, and complex conjugation, respectively. The modulus of a complex scalar is written as |a|, the Euclidean norm of a vector as ${∥a∥}_{2}$, and the Frobenius norm of a matrix as ${∥A∥}_{F}$. The scalar ${\left[a\right]}_{q}$ is entry *q* of vector a; entrywise magnitude comparisons use ${|\left[a\right]}_{q}|$. The phase of a nonzero complex scalar is arg(a). For the geometric illustrations, a→=(Rea,Ima)T represents the corresponding vector in the real plane, and $\vec{a}\;\cdot \;\vec{b}$ is its Euclidean inner product. In particular, $h_{k}$ and $h_{k,i}$ are channel vectors, $h_{k,q}={\left[h_{k}\right]}_{q}$ is a scalar channel coefficient, $H_{e}$ is the effective-channel matrix, and ${\hat{h}}_{k}$ is an estimated channel vector.

## 2. Related Work

This section reviews hybrid architectures, discrete analog-state searches, antenna selection, and learning-assisted processing. It includes recent developments relevant to computational cost and power consumption, and concludes by distinguishing their design variables and objectives from the proposed fixed-subarray SI method.

### 2.1. Hybrid Architectures and Subarray Design

Phase-shifter-based hybrid precoding provides adjustable spatial weights with fewer RF chains than a fully digital array. Representative designs exploit sparse mmWave channels to approximate a desired digital precoder [4], optimize analog and digital weights under phase constraints [6], or alternate between the two stages [9]. Their hardware and CSI requirements are summarized in the hybrid-beamforming survey [5]. Fully connected networks offer more analog design freedom, whereas fixed sub-connected networks restrict each RF chain to one antenna subset [10,11,12]. Low-resolution phase shifters reduce component cost and power but further restrict the available weights [13,14]. Wideband designs additionally use one analog network across subcarriers with subcarrier-dependent digital processing [15,16]; this joint frequency-domain objective differs from the local frequency-flat SI gain considered here. Power consumption also depends on the RF chains and converter resolution, as examined in [17].

Yuan et al. [18] studied point-to-point mmWave hybrid precoding and combining with a two-layer low-resolution phase-shifter network. They proposed a two-level codebook, adaptive-weighted cross-entropy search, and greedy gain-based alternatives to limit search work. Their approach changes the phase-shifter network and codebook design; the present SI architecture instead uses direct and inverted signal paths with an optional inactive state.

Sub-connected architectures can use either fixed or dynamic antenna-to-RF-chain mappings. With a fixed mapping, each RF chain serves a predetermined antenna subset; a dynamic mapping adapts these subsets to the channel conditions [11,19]. Magueta et al. [19] proposed a hybrid multi-user equalizer for broadband uplink mmWave massive MIMO with a dynamic sub-connected architecture. Their design combines per-subcarrier digital equalization with an analog equalizer shared across subcarriers and sequentially selects antenna-to-RF-chain connections and quantized phase shifts. In the present work, the subarray connections are fixed. We address downlink hybrid precoding by optimizing switch and inverter states and selecting active antennas within these subarrays, without reallocating antennas among RF chains.

Zhao et al. [20] developed channel-hardening-aided two-stage precoding for sub-connected mmWave multi-user massive MIMO. An approximate effective-channel formulation supports closed-form analog design and energy-efficient digital processing, with an additional subarray/user-assignment mechanism. These phase-shifter and assignment decisions differ from the fixed antenna-to-RF-chain topology and discrete SI-state comparisons used here.

Oh and Choi [21] exploit a low-dimensional subspace property for phase-shifter hybrid precoding, with reduced-complexity digital optimization. Their framework also supports dynamic antenna partitioning and covariance-based statistical CSI. This provides a recent example of reducing optimization dimensions and adapting connections; our design keeps the connections fixed and operates on complex partial sums of discrete SI coefficients.

### 2.2. Switch-Based Processing and Antenna Selection

Switch-based architectures provide an alternative to phase-shifter networks when hardware cost and power consumption are important [7]. In SI precoding, cross-entropy sampling [22] evaluates complete analog configurations, whereas coordinate updates [23] repeatedly test individual states with the other states fixed. SI hybrid designs have also been studied with generalized spatial modulation [24]. Their different objectives and search variables must be considered when comparing computational workload and achieved performance.

Antenna selection provides a complementary way to adjust the rate–power trade-off by activating only part of an array. Representative methods use greedy subset selection [25], convex approximations for joint beamforming and selection [26], or adaptive cross-entropy searches [27]. Decremental selection has also been incorporated into hybrid combining with active/inactive switches [28].

Shen et al. [29] proposed message-passing antenna and RF-chain selection for a partially connected receiving hybrid beamformer whose connections follow an LDPC-inspired structure. Sequential and parallel variants address selection and convergence. This receiver-side joint antenna/RF-chain selection differs from the transmitter-side SI method studied here, which retains the RF-chain mapping and deactivates antenna paths under analog-gain constraints.

### 2.3. Learning-Assisted and Other Multi-User Designs

Learning-assisted analog beamforming, such as the reinforcement-learning design in [30], provides a different mechanism based on a trained decision policy; its training requirements and design constraints differ from these direct partial-sum comparisons.

Multi-user precoding also arises in RIS-assisted transmission. Castillo-Soria et al. [31] studied a reconfigurable intelligent surface (RIS)-assisted multi-user MIMO system with double quadrature spatial modulation (DQSM). Their base-station precoding strategies address interference along the BS-to-RIS and RIS-to-user links, and are evaluated in terms of bit error rate and detection complexity. The present study focuses on the fixed sub-connected SI hybrid architecture at the base station and examines the trade-off among sum rate, power consumption, and the computation required for discrete analog-state selection and antenna activation.

### 2.4. Position of the Proposed Method

The reviewed approaches reduce computational or hardware costs through channel approximations, constrained codebooks, reduced-dimensional processing, adaptive connections, message passing, or trained decision policies. These mechanisms address different feasible sets and processing objectives.

The present method combines sequential selection of SI analog states with antenna deactivation under QoS constraints. A native DP-based procedure reuses complex partial sums; amplitude ordering and additional candidate comparisons are then introduced to mitigate the effect of discarding intermediate states. The deactivation stage uses beamforming-amplitude contribution estimates and re-evaluates affected decisions while seeking to retain prescribed analog gains. The mechanism and workload comparison with CE and CU is developed in Section 5.3. The proposed procedure remains suboptimal in general, and neither its local gain objective nor antenna-count surrogate establishes global sum-rate or EE maximization.

Table 1 summarizes representative approaches and their relation to this work. The numerical SI benchmarks are CE and CU under the specified settings; phase-shifter-based and fully digital schemes are architecture references. Dynamic-subarray, wideband, combining, and learning-assisted studies supply methodological comparisons across different design variables and assumptions.

## 3. System Model

We adopt a frequency-flat narrowband sparse geometric channel model for the mmWave multi-user downlink. The occupied signal bandwidth is taken to be small relative to the channel coherence bandwidth, and the frequency variation of the array response over that bandwidth is neglected. If *B* denotes the occupied bandwidth, Δτ the propagation-path excess-delay spread, and $\tau_{\mathrm{ap}}$ the maximum differential propagation delay across the array, the small-variation conditions BΔτ≪1 and $B\tau_{\mathrm{ap}}\ll 1$ provide sufficient conditions for these approximations. These conditions define the frequency-flat approximation adopted in the analysis. This choice permits a focused analysis of spatial switch-inverter state selection, sequential DP updates, and antenna deactivation under a QoS constraint, while retaining spatial multipath and inter-user interference. The relationship to wideband transmission is discussed in Section 4.5.

**Figure 1:**
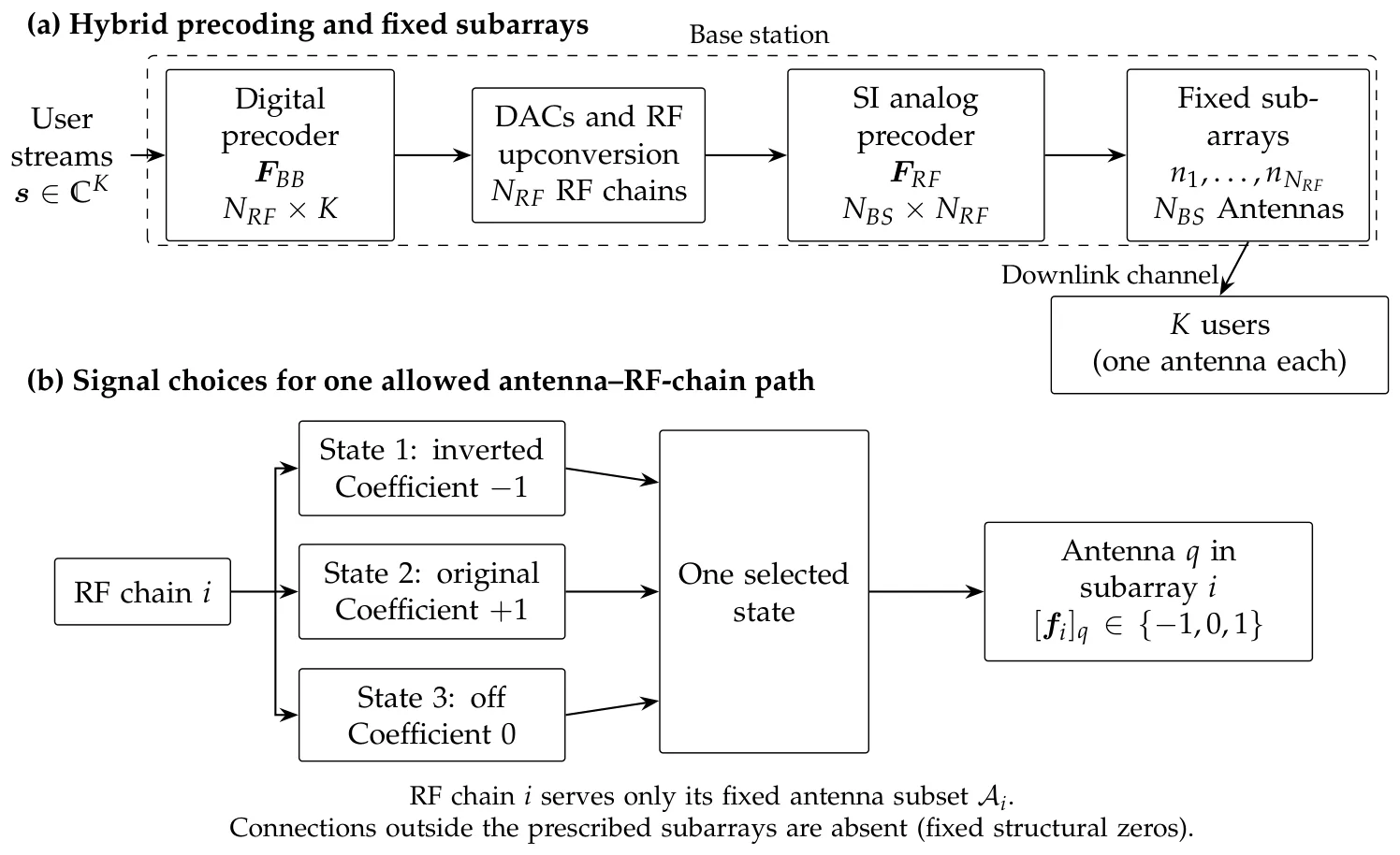
Switch-inverter (SI) hybrid precoding: (**a**) the digital-to-RF-to-antenna processing chain with fixed subarrays; (**b**) alternative signal states for one allowed antenna–RF-chain path. Exactly one state is selected: inverted (−1), original (+1), or inactive (0). The off-subarray connections are fixed structural zeros.

In this paper, a mmWave massive MIMO system is considered, where a base station (BS) is equipped with $N_{BS}$ antennas ($N_{BS}\gg 1$) serves *K* single-antenna mobile stations (MSs) simultaneously. The BS employs a hybrid architecture, where a fully digital baseband precoder $F_{BB}\in C^{N_{RF}\times K}$ and an analog precoder $F_{RF}\in C^{N_{BS}\times N_{RF}}$ are jointly employed, connected via $N_{RF}$ RF chains. Each MS is served by one data stream, and the general formulation allows $K\leq N_{RF}\leq N_{BS}$. Supporting *K* simultaneous streams with the adopted ZF precoder additionally requires the effective channel to have full row rank. The numerical evaluation uses $N_{RF}=K$, the minimum RF-chain count compatible with *K* independent streams under this rank condition; this is a simulation setting rather than a requirement of the general model. Different from a traditional analog precoder that uses high-resolution PSs, the proposed architecture uses energy efficient switches and inverters with a sub-connected architecture. As shown in Figure 1, RF chain *i* is connected to a fixed antenna subset $A_{i}$ of size $n_{i}$. The subsets are disjoint and cover the array, so $\sum_{i=1}^{N_{RF}}n_{i}=N_{BS}$. Equal-size subarrays are the special case $n_{i}=N_{BS}/N_{RF}$ when this ratio is an integer. The switch determines the status of the corresponding antenna: when the switch turns to state 1, the input signal is inverted; state 2 means that the original signal is sent to the antenna; state 3 indicates that the antenna is switched off. This switch-inverter (SI) analog precoder is very energy efficient compared to the high-resolution PS precoder, since the energy consumption for one switch ($P_{SW}=5$ mW [32]) or inverter ($P_{IN}\approx P_{SW}$ [22]) is far less than a high-resolution PS ($P_{PS}=40$ mW for a 4-bit PS [32]), while offering the discrete analog-state design studied in this work. Its performance depends on the channel, RF-chain configuration, and state-selection procedure; the resulting rate–power trade-off is evaluated in Section 6.

The digital precoder $F_{BB}$ maps the *K* user data streams to the $N_{RF}$ RF-chain signals, and the analog precoder $F_{RF}$ maps those signals to the $N_{BS}$ antennas. The product $F_{RF}F_{BB}$ is the overall hybrid precoder; its *k*th column contains the effective transmit beamforming weights for user *k*. Thus, both stages contribute to the spatial weighting.

We use the fixed-subarray SI structure in [22] (Equations (7) and (8)), allowing unequal block sizes, unscaled hardware coefficients, and the additional off state for antenna deactivation. The analog precoder is the block-diagonal matrix $F_{RF}=\mathrm{diag}\left[f_{1},\ldots ,f_{N_{RF}}\right]$, where $f_{i}\in C^{n_{i}\times 1}$ and $i=1,\ldots ,N_{RF}$. Each block satisfies

(1) $${\left[f_{i}\right]}_{q}\in \left\{-1,0,1\right\},\;q=1,\ldots ,n_{i}.$$

These are the unscaled SI hardware coefficients. A value of −1 denotes an active antenna transmitting the phase-inverted signal, equivalent to a phase shift of π (switch state 1); 0 denotes an antenna switched off (switch state 3); and 1 denotes an active antenna transmitting the original signal (switch state 2). These controllable coefficients are the entries of the block vectors $f_{i}$. Entries of $F_{RF}$ outside the prescribed block structure are fixed zeros corresponding to absent antenna–RF-chain connections; they are not antenna-deactivation variables. Transmit-power normalization is specified separately below.

**Figure 2:**
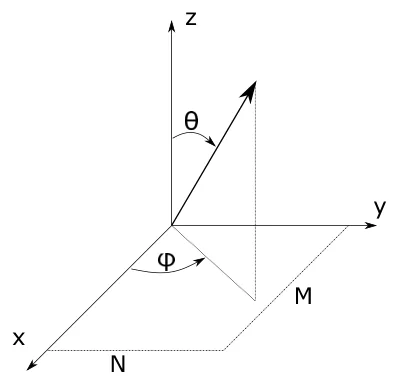
Geometric structure of the uniform planar array (UPA) in the xy-plane. The polar angle θ is measured from the positive *z*-axis (the array normal), and the azimuth ϕ from the positive *x*-axis in the array plane.

We assume that the BS is equipped with a uniform planar array (UPA) with $M\times N=N_{BS}$ antennas in the xy-plane. The uniform linear array (ULA) case is obtained by setting N=1. We adapt the UPA direction-cosine representation of El Ayach et al. [4] (Section II-B) to this array plane and our indexing convention. As shown in Figure 2, θ∈(0,π/2) is the polar angle from the positive *z*-axis, and ϕ∈(0,2π) is the azimuth from the positive *x*-axis. Element (m,n) is located at ((m−1)d,(n−1)d,0). With unit-modulus entries, the steering vector is

(2) $$\begin{array}{cc} a\left(\theta ,\phi \right)= & \left[1,\cdots ,e^{j\frac{2\pi d}{\lambda }\left[\left(m-1\right)\sin\theta \cos\phi +\left(n-1\right)\sin\theta \sin\phi \right]}\right.,\\ & {\left.\cdots ,e^{j\frac{2\pi d}{\lambda }\left[\left(M-1\right)\sin\theta \cos\phi +\left(N-1\right)\sin\theta \sin\phi \right]}\right]}^{T}, \end{array}$$

where *d* is the inter-element spacing and λ is the wavelength. Our response is unnormalized, with ${∥a\left(\theta ,\phi \right)∥}_{2}^{2}=N_{BS}$, whereas the cited representation uses unit-norm array responses. Following the finite-path sparse geometric representation in [4] (Equation (4)), we use its single-antenna-receiver form with the path-gain scaling specified here:

(3) $$\begin{array}{c} h_{k}=\sum_{l=1}^{L_{k}}g_{k,l}a\left(\theta_{k,l},\phi_{k,l}\right), \end{array}$$

where $L_{k}$ is the number of propagation paths, $\left\{\theta_{k,l},\phi_{k,l}\right\}$ the Direction of Departure (DoD) of the *l*th path, and $g_{k,l}$ the corresponding complex channel gain. Equation (3) has no additional path-count normalization factor; the path-gain distribution and path counts used here are specified. These are our scaling conventions, rather than a direct copy of the normalized clustered-channel parameters in the cited model.

We use the standard multi-user hybrid received-signal framework of Gao et al. [22] (Equation (1)), with the following equal-power and column-normalization conventions. Let $s_{k}$ denote the unit-variance data symbol for MS *k*, with independent symbols across users. With equal stream power ρ/K and normalized hybrid-precoder columns as specified below, the received signal is

(4) $$\begin{array}{c} y_{k}=\sqrt{\frac{\rho }{K}}\;h_{k}^{H}F_{RF}{\left[F_{BB}\right]}_{:,k}s_{k}+\sqrt{\frac{\rho }{K}}\sum_{u\neq k}^{K}h_{k}^{H}F_{RF}{\left[F_{BB}\right]}_{:,u}s_{u}+z. \end{array}$$

where $z∼\mathrm{CN}(0,\sigma^{2})$ is additive Gaussian noise. The first term is the desired signal and the sum is inter-user interference. For a given analog precoder, the effective channel stacks the users’ channel rows:

$$H_{e}=\left[\begin{array}{c} h_{1}^{H}F_{RF}\\ \vdots \\ h_{K}^{H}F_{RF} \end{array}\right]\in C^{K\times N_{RF}}.$$

The steering-vector construction in (3) induces spatial structure and dependence among antenna coefficients; they are not modeled as independent Rayleigh entries. The scalar DP comparisons operate on the given complex coefficients without requiring statistical independence. For multi-user ZF, the relevant property is instead linear independence of the rows of $H_{e}$. Independent statistical parameter generation alone does not guarantee this property after analog projection. Distinct spatial directions can aid user separability, but the selected SI weights also determine the effective-channel rank and conditioning. The gain constraints used for antenna deactivation do not by themselves guarantee full row rank. The ZF formulation, therefore, applies to retained configurations satisfying the rank condition below, including after deactivation; poorly conditioned effective channels can reduce the achievable rate after transmit-power normalization.

If $\mathrm{rank}(H_{e})=K$, the standard effective-channel ZF pseudoinverse [22] (Equation (15)) gives the unnormalized digital precoder

$${\tilde{F}}_{BB}=H_{e}^{H}{\left(H_{e}H_{e}^{H}\right)}^{-1}\in C^{N_{RF}\times K}.$$

The matrix $H_{e}H_{e}^{H}$ is then invertible even when $K<N_{RF}$. The cited formulation uses a common Frobenius-norm scaling; for the equal-stream-power convention in (4), we instead normalize each hybrid-precoder column according to

$${\left[F_{BB}\right]}_{:,k}=\frac{{\left[{\tilde{F}}_{BB}\right]}_{:,k}}{∥F_{RF}{\left[{\tilde{F}}_{BB}\right]}_{:,k}{∥}_{2}}.$$

Thus, $∥F_{RF}{\left[F_{BB}\right]}_{:,k}{∥}_{2}=1$ and the total transmit power is ρ. The rank condition must hold for the retained analog configuration, including after antenna deactivation. The inequality $K\leq N_{RF}$ alone does not guarantee it. Using the standard SINR and achievable sum-rate expressions [22] (Equations (13) and (14)) with our power convention, and with the superscript ∗ denoting the selected precoders, the SINR is

(5) $$\begin{array}{c} {\mathrm{SINR}}_{k}=\frac{\frac{\rho }{K}{\left|h_{k}^{H}F_{RF}^{*}{\left[F_{BB}^{*}\right]}_{:,k}\right|}^{2}}{\sigma^{2}+\sum_{u\neq k}^{K}\frac{\rho }{K}{\left|h_{k}^{H}F_{RF}^{*}{\left[F_{BB}^{*}\right]}_{:,u}\right|}^{2}}, \end{array}$$

where ρ is the transmitting power, and is evenly allocated to every MS. The overall achievable sum-rate R (bit/Hz/s) is given by

(6) $$\begin{array}{c} R=\sum_{k=1}^{K}\log_{2}\left(1+{\mathrm{SINR}}_{k}\right). \end{array}$$

Following the rate-to-power EE definition in [22] (Section IV), EE measures achievable aggregate throughput per unit total consumed power. Since *R* in (6) is the achievable sum rate per unit bandwidth, the EE reported throughout this manuscript is likewise bandwidth-normalized:

$$\xi ≜\frac{R}{P_{all}}.$$

With *R* in bit/s/Hz and $P_{all}$ in watts, ξ is in bit/s/Hz/W, equivalently bit/J/Hz. For a fixed occupied bandwidth *B*, the aggregate data rate is $R_{\mathrm{data}}=BR$ in bit/s, and the corresponding EE is $R_{\mathrm{data}}/P_{all}=B\xi$ in bit/J. Multiplication by the same *B* preserves all EE comparisons. Active-antenna count is a hardware-state quantity affecting both the achievable rate and circuit power; ξ evaluates the resulting rate-to-power ratio. Following the component-based hardware accounting in [22] (Equation (6)), we adapt the total-power model to the active branches of our fixed-subarray SI architecture:

(7) $$\begin{array}{c} P_{all}=P_{BB}+N_{RF}P_{RF}+\rho +N_{BS}^{A}P_{SW}+N_{BS}^{I}P_{IN}, \end{array}$$

where $P_{all}$ is the overall power consumption, and $P_{RF}$, $P_{SW}$, and $P_{IN}$ are the power consumption of one RF chain, switch, and inverter, respectively, and ρ is the total transmit power under the normalization in (4); the normalized hybrid precoder satisfies $∥F_{RF}F_{BB}{∥}_{F}^{2}=K$. Here $N_{BS}^{A}\leq N_{BS}$ denotes the number of “active” antennas, and $N_{BS}^{I}\leq N_{BS}^{A}$ is the number of “inverted” antennas. An antenna is active when its corresponding switch is turned to state 1 (inverted signal) or state 2 (original signal), which means that the antenna is working. An antenna is called an inverted antenna when its corresponding switch is turned to state 1 (inverted signal). The cited SI model counts one inverter per RF chain and switches for all antennas. In (7), we instead assume one switch-power contribution per active antenna and one inverter-power contribution per inverted antenna; inactive branches contribute neither term. The baseband, RF-chain, and transmit-power terms remain fixed during antenna deactivation. This counting is specific to the SI architecture and power assumptions used here.

## 4. SI Based Hybrid Precoding Scheme

### 4.1. Analog Beamforming-Gain Objective

To design the analog precoder for the SI architecture, we first consider the case where all antennas are active. Each controllable coefficient within a connected subarray is then a two-valued sign variable. The hardware constraint in (1) becomes

(8) $${\left[f_{i}\right]}_{q}\in \left\{-1,1\right\},\;q=1,\ldots ,n_{i},\;i=1,\ldots ,N_{RF}.$$

This restriction applies to the entries of the block vectors $f_{i}$. Any structural zeros outside those blocks remain fixed when all antennas are active. For a given analog precoder with a full-row-rank effective channel, the ZF digital precoder can be computed as described in Section 3. We, therefore, consider the analog-gain design assuming perfect CSI. For a fixed user *k*, a userwise gain objective is

(9) $$F_{RF,k}^{★}\in \underset{F_{RF}}{\arg\max}{∥h_{k}^{H}F_{RF}∥}_{2}^{2}.$$

The hardware constraint is (8). Let $h_{k,i}$ be user *k*’s channel restricted to $A_{i}$. The block-diagonal architecture gives

$$∥h_{k}^{H}F_{RF}{∥}_{2}^{2}=\sum_{i=1}^{N_{RF}}{\left|h_{k,i}^{H}f_{i}\right|}^{2}.$$

For this fixed-user gain objective and independent block constraints, the sign choices can be considered block by block. The full array has $N_{BS}$ binary sign variables and $2^{N_{BS}}$ possible patterns, while block *i* has $2^{n_{i}}$ patterns. This decomposition motivates the local DP comparison below. Different users generally prefer different signs, so (9) is a userwise design objective rather than a simultaneous maximizer of every user’s gain or of the multi-user sum rate. The local recursion does not by itself specify a general user-to-subarray coordination policy for $K<N_{RF}$; the reported system-level performance is evaluated for $K=N_{RF}$.

### 4.2. Sequential DP Design of the Analog Precoder

For SI analog-precoder design, previous methods include iterative heuristic search [22,33] and coordinate updates that hold the other signs fixed [23]. Related joint antenna-selection and low-resolution analog-precoder design is considered in [34]. For the sequential procedure proposed here, we use comparisons of complex partial sums. The scalar analysis concerns a fixed user and a fixed subarray; it must not be interpreted as a coherent sum across different RF-chain outputs.

For a chosen pair (k,i), define $c_{q}=h_{k,q}^{*}$ for $q\in A_{i}$ and $c_{q}=0$ otherwise. This zero-padded coefficient vector lets us retain the full-array antenna indexing in the scalar recurrence:

(10) $$c={\left[\alpha_{1}e^{j\gamma_{1}}\;\alpha_{2}e^{j\gamma_{2}}\;\cdots \;\alpha_{N_{BS}}e^{j\gamma_{N_{BS}}}\right]}^{T}.$$

**Figure 3:**
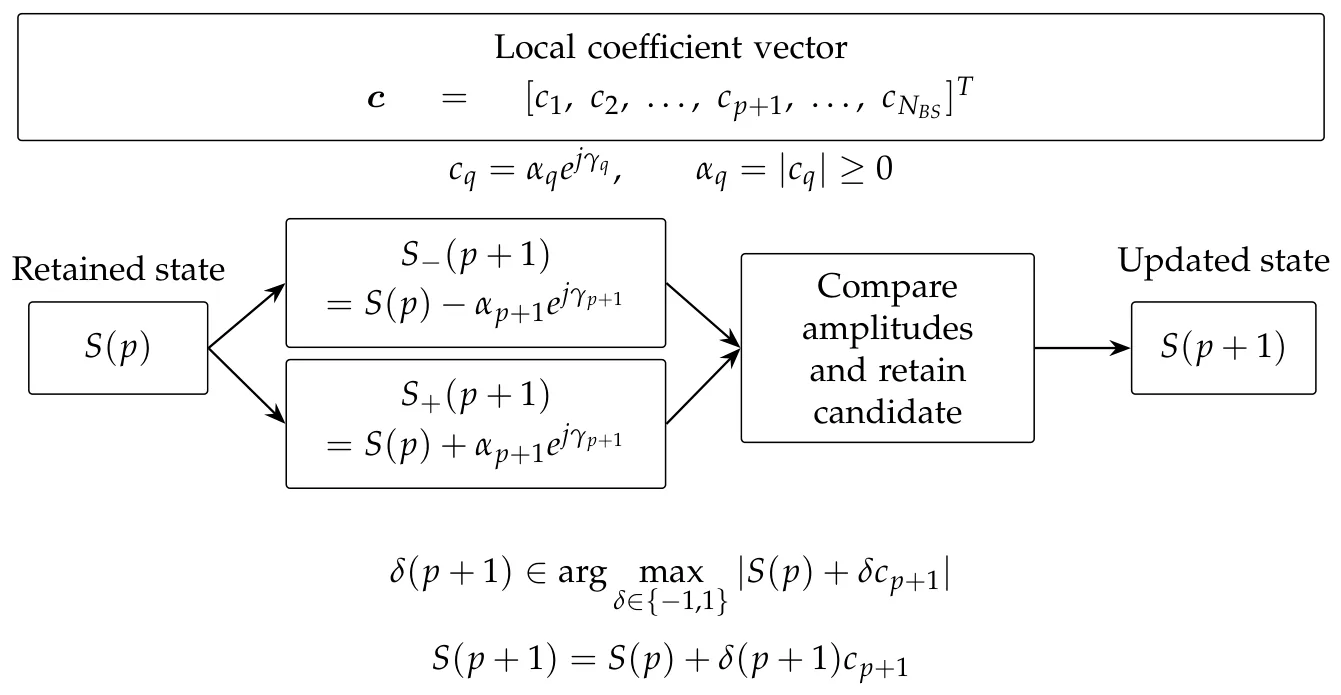
The native DP update for the local coefficient vector c. The scalar coefficient is $c_{q}=\alpha_{q}e^{j\gamma_{q}}$ with $\alpha_{q}=\left|c_{q}\right|$. The negative- and positive-sign candidates are $S_{-}\left(p+1\right)$ and $S_{+}\left(p+1\right)$; the retained complex state is S(p+1).

Here $\alpha_{q}=\left|c_{q}\right|\geq 0$ is a scalar magnitude and $\gamma_{q}=\arg\left(c_{q}\right)$ is its phase when $c_{q}\neq 0$; the phase of a zero coefficient is immaterial. Greek amplitude and phase symbols are ordinary mathematical italic scalars, while c is a vector. Only antenna states within $A_{i}$ are varied in this local update, and the other blocks are held fixed. Substituting these definitions into the local gain gives

(11) $$\begin{array}{c} {\left|h_{k,i}^{H}f_{i}\right|}^{2}={\left|\sum_{q=1}^{N_{BS}}\delta \left(q\right)c_{q}\right|}^{2}={\left|\sum_{q\in A_{i}}\delta \left(q\right)c_{q}\right|}^{2}. \end{array}$$

where δ(q)∈{−1,1} selects the switch state of antenna *q* within the chosen subarray: −1 denotes an inverted signal and 1 a direct signal. The contribution from outside that subarray is zero in (11), regardless of the fixed states there. The scalar DP selects candidate signs for this local gain. Zero entries may be omitted in implementation, in which case the sequence length is $n_{i}$. The local examples and schematics below describe this coefficient sequence. Figure 3 explicitly label the local vector as c, distinguishing it from the physical channel $h_{k}$.

For the proposed sequential procedure, define the scalar state $S\left(p\right)=\sum_{q=1}^{p}\delta \left(q\right)c_{q}$ as the retained partial sum for the chosen local coefficient sequence. At the next coefficient $c_{p+1}=\alpha_{p+1}e^{j\gamma_{p+1}}$, the native DP compares the two possible signs while keeping the preceding signs fixed. Directly adding the next signed coefficient gives the following candidates for $p=1,\ldots ,N_{BS}-1$ in the padded indexing:

(12) $$\begin{array}{cc} S_{-}\left(p+1\right) & =S\left(p\right)-\alpha_{p+1}e^{j\gamma_{p+1}}, \end{array}$$

(13) $$\begin{array}{cc} S_{+}\left(p+1\right) & =S\left(p\right)+\alpha_{p+1}e^{j\gamma_{p+1}}. \end{array}$$

The candidates $S_{-}\left(p+1\right)$ and $S_{+}\left(p+1\right)$ correspond to δ(p+1)=−1 and 1, respectively. The candidate with the larger amplitude is retained as S(p+1); an equal-amplitude comparison permits either candidate under the chosen tie-breaking rule. Thus, (12) and (13) define alternative candidates, rather than two simultaneous values of the retained state. Outside the chosen subarray, $c_{p+1}=0$ and the physical antenna state is held fixed. After omitting zero entries and relabeling the local sequence, its first sign can be fixed to 1 because reversing every local sign leaves the local gain unchanged. The native procedure then requires $n_{i}-1$ binary candidate comparisons. Figure 3 illustrates the transition from S(p) to S(p+1).

### 4.3. Optimality Analysis

The proposed DP-based approach updates a retained complex partial sum sequentially. We call this the native DP scheme. For a fixed retained state S(p), the next sign is selected exactly within the two available candidates:

$$\delta_{p+1}\in \arg\max_{\delta \in \{-1,1\}}{\left|S\left(p\right)+\delta c_{p+1}\right|}^{2}.$$

This conditional comparison does not imply global optimality over all sign sequences. Expanding the candidate squared magnitude gives the identity

|S+δc|2=|S|2+|c|2+2δRe(S*c),

so the continuation depends on the phase of the retained partial sum as well as its amplitude. A state discarded because of its smaller current amplitude may yield a larger final amplitude after subsequent coefficients are added. The following examples explain this limitation and motivate the revised scheme.

**Figure 4:**
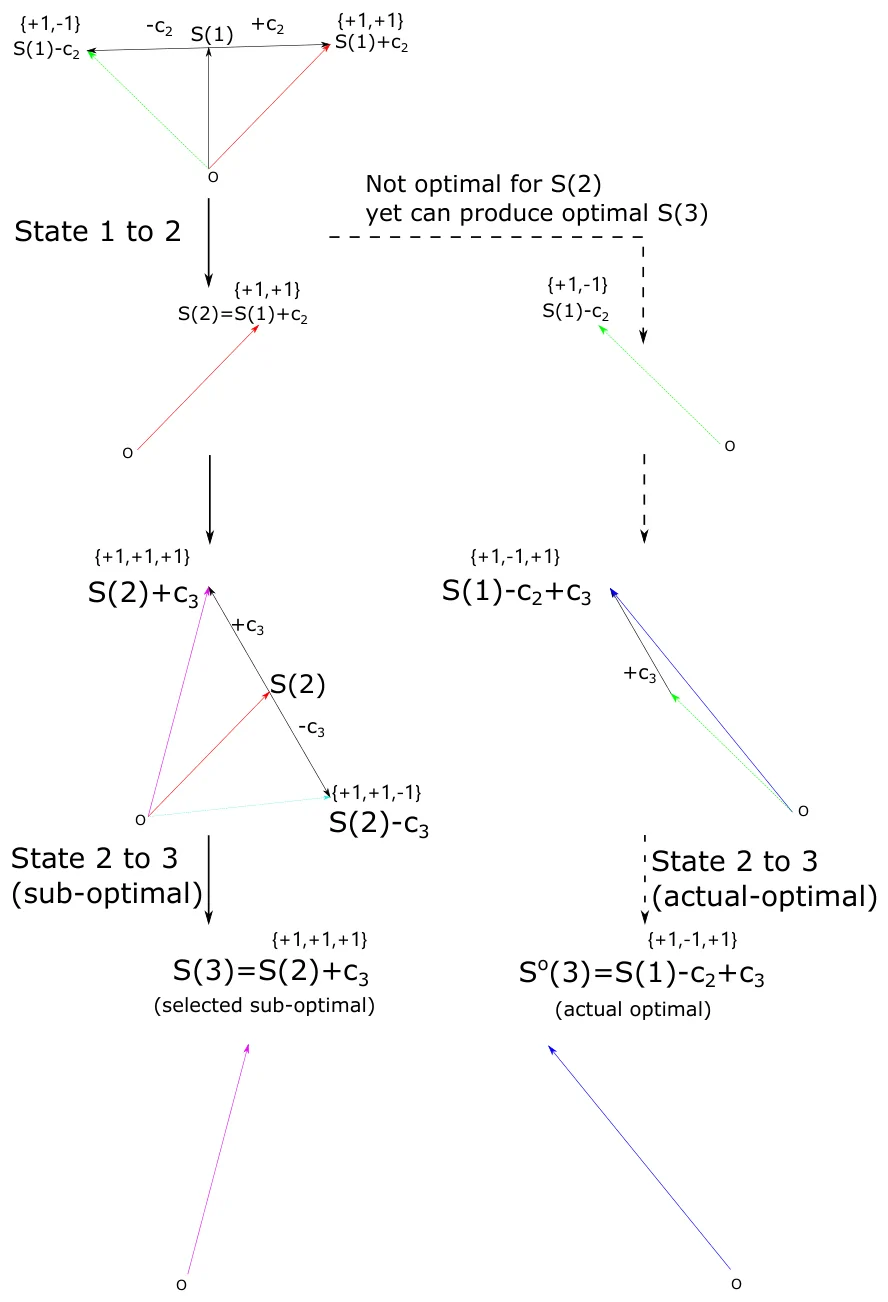
An example of sub-optimality of the DP process.

Denoting the actual optimal state with largest amplitude as $S^{o}\left(i\right)$, it is apparent that the first and second state calculated with the native DP is already optimal (i.e., $\left|S\left(i\right)|=\right|S^{o}\left(i\right)|,i=1,2$). For the third state, assuming that the optimal selecting functions $\left\{1,1\right\}$ have been selected for S(2), it tests the amplitudes of selecting functions $\left\{1,1,-1\right\}$ and $\left\{1,1,1\right\}$ and selects the one with larger amplitude. We then assume that $\left\{1,1,1\right\}$ is selected. However, there does exist the possibility that $\left\{1,-1,1\right\}$ will provide a larger amplitude. Since $\left\{1,1\right\}$ has larger amplitude than $\left\{1,-1\right\}$ and is kept for State 2, the actual optimal $\left\{1,-1,1\right\}$ cannot be taken into consideration based on $\left\{1,1\right\}$, causing a sub-optimality for the DP process. This example of sub-optimality is demonstrated in Figure 4, where $c_{q}=\alpha_{q}e^{j\gamma_{q}}$ denotes a coefficient of the local sequence in (10). Evidently, the sub-optimality will take place when the amplitudes of $c_{1}+c_{2}$ and $c_{1}-c_{2}$ are very close to each other, while the direction of $c_{3}$ is close enough to $c_{1}-c_{2}$, so that $\left|c_{1}-c_{2}+c_{3}\right|>\left|c_{1}+c_{2}+c_{3}\right|$.

**Lemma** **1.**  *For fixed magnitudes $\left|S\left(p-1\right)\right|=\alpha_{S}$ and $|c_{p}|=\alpha_{p}$, the amplitude of the next state selected by the native DP is minimized over the relative phase when S(p−1) and $c_{p}$ are perpendicular.*

**Proof.**  Denoting $S\left(p-1\right)$ as $S\left(p-1\right)=\alpha_{S}e^{j\gamma_{S}}$, we have

(14) $$\begin{array}{cc} \; & {\left|S\left(p\right)\right|}^{2}\\ & ={\left(\alpha_{S}\cos\gamma_{S}\mp \alpha_{p}\cos\gamma_{p}\right)}^{2}+{\left(\alpha_{S}\sin\gamma_{S}\mp \alpha_{p}\sin\gamma_{p}\right)}^{2}\\ & ={\alpha_{S}}^{2}+{\alpha_{p}}^{2}\mp 2\alpha_{S}\alpha_{p}\left(\cos\gamma_{S}\cos\gamma_{p}+\sin\gamma_{S}\sin\gamma_{p}\right)\\ & ={\alpha_{S}}^{2}+{\alpha_{p}}^{2}\mp 2\alpha_{S}\alpha_{p}\cos\left(\gamma_{S}-\gamma_{p}\right), \end{array}$$

After selecting the better of the two signs in (14), the squared amplitude is $\alpha_{S}^{2}+\alpha_{p}^{2}+2\alpha_{S}\alpha_{p}\left|\cos\left(\gamma_{S}-\gamma_{p}\right)\right|$. For fixed $\alpha_{S}$ and $\alpha_{p}$, its maximum is ${\left(\alpha_{S}+\alpha_{p}\right)}^{2}$ when the vectors are collinear, and its minimum is $\alpha_{S}^{2}+\alpha_{p}^{2}$ when they are perpendicular. Consequently, the selected amplitude lies in $\left[\sqrt{\alpha_{S}^{2}+\alpha_{p}^{2}},\alpha_{S}+\alpha_{p}\right]$. These bounds concern a single update conditioned on a retained state.

To provide a mathematical instance, we consider an extreme case where $\alpha_{1}=\alpha_{2}=\alpha_{3}=1$, $\gamma_{1}=0$, $\gamma_{2}\to \pi /2^{-}$, $\gamma_{3}=-\pi /4$. Here $\gamma_{2}\to \pi /2^{-}$ indicates that $\gamma_{2}\to \pi /2$ and $\gamma_{2}<\pi /2$ which guarantees that $\left|c_{1}+c_{2}\right|>\left|c_{1}-c_{2}\right|$. Then the corresponding states S(1), S(2), S(3) and the actual optimal $S^{o}\left(3\right)$ are computed as   

(15) $$\begin{array}{cc} \; & S\left(1\right)=c_{1}=1,\;S\left(2\right)=S\left(1\right)+c_{2}\to \sqrt{2}e^{j\pi /4},\\ & S\left(3\right)=S\left(2\right)+c_{3}\to \left(1+\frac{\sqrt{2}}{2}\right)+j\left(1-\frac{\sqrt{2}}{2}\right)\\ & S^{o}\left(3\right)=c_{1}-c_{2}+c_{3}\to \left(1+\frac{\sqrt{2}}{2}\right)-j\left(1+\frac{\sqrt{2}}{2}\right), \end{array}$$

and amplitudes $\left|S(3)\right|$ and $\left|S^{o}\left(3\right)\right|$ are

(16) $$\begin{array}{cc} \; & \left|S(3)\right|=\sqrt{{\left(1+\frac{\sqrt{2}}{2}\right)}^{2}+{\left(1-\frac{\sqrt{2}}{2}\right)}^{2}}=\sqrt{3}\\ & \left|S^{o}\left(3\right)\right|=\sqrt{{\left(1+\frac{\sqrt{2}}{2}\right)}^{2}+{\left(1+\frac{\sqrt{2}}{2}\right)}^{2}}=\sqrt{2}+1. \end{array}$$

In this limiting example, the amplitude loss relative to the optimum is $1-\frac{\sqrt{3}}{\sqrt{2}+1}\approx 28.26\%$. This case demonstrates that a locally preferred intermediate state can exclude the globally best continuation and motivates additional candidate comparisons. It does not establish a general worst-case loss.    □

### 4.4. Revised DP Design of the Analog Precoder

In the trajectory comparisons below, $S^{o}\left(q\right)$ denotes the partial sum at stage *q* along a sign sequence that maximizes the final amplitude for the complete coefficient sequence. An intermediate $S^{o}\left(q\right)$ need not maximize the amplitude of the truncated sequence by itself. Thus, even if $S\left(p\right)=S^{o}\left(p\right)$, the retained trajectory can satisfy $\left|S\left(p+1\right)|>\right|S^{o}\left(p+1\right)|$ but $\left|S\left(p+2\right)|<\right|S^{o}\left(p+2\right)|$. The two trajectories then differ in their sign choices after stage *p*.

One illustrative configuration involves intermediate states with similar amplitudes but different phases. By Lemma 1, the next selected amplitude from a state of magnitude *a* and a coefficient of magnitude α lies between $\sqrt{a^{2}+\alpha^{2}}$ and a+α. A smaller intermediate amplitude can, therefore, be offset by a more favorable relative phase at a later update.

For example, the added coefficient may be aligned with the discarded state and nearly perpendicular to the retained state, as illustrated by

(17) $$\left\{\begin{array}{c} \left|∠\left\{S^{o}\left(p+1\right)\right\}-∠\left\{c_{p+2}\right\}\right|\to 0\\ \left|∠\left\{S\left(p+1\right)\right\}-∠\left\{c_{p+2}\right\}\right|\to \pi /2 \end{array}\right.,$$

so that the continuation from $S^{o}\left(p+1\right)$ can exceed that from S(p+1). This is an illustrative mechanism, rather than a necessary or exhaustive characterization of suboptimality.

Therefore, compared to the native DP demonstrated in (12) and (13) the revised DP involves the two following revisions:

1. The local coefficients $c_{q}$ in c are sorted in descending order of amplitude. The sorted local coefficient vector is denoted by $r_{k}={\left[r_{1},\ldots ,r_{N_{BS}}\right]}^{T}$, with corresponding antenna indices $b_{1},\ldots ,b_{N_{BS}}$; its entries $r_{q}$ are complex scalars. This processes larger coefficients first. For a fixed partial sum, a smaller added coefficient has a smaller possible effect on its amplitude. This motivates the ordering, but does not provide a global optimality guarantee or a probability bound on suboptimality.

2. The revised DP compares both signs of the next two coefficients for a retained prefix S(p). Substituting the four sign pairs into the partial-sum definition gives the candidate amplitudes compared at the update for antenna p+2:

(18) $$\begin{array}{c} \left\{\begin{array}{c} \left|S\left(p\right)+r_{p+1}+r_{p+2}\right|\\ \left|S\left(p\right)+r_{p+1}-r_{p+2}\right|\\ \left|S\left(p\right)-r_{p+1}+r_{p+2}\right|\\ \left|S\left(p\right)-r_{p+1}-r_{p+2}\right| \end{array}\right., \end{array}$$

and a candidate with the largest amplitude is selected. The first two candidates use the prefix $S\left(p\right)+r_{p+1}$, whereas the other two use $S\left(p\right)-r_{p+1}$. Thus, the sign of antenna p+1 can be reconsidered when antenna p+2 is processed. This comparison is exact over the four candidates conditioned on S(p); signs already fixed within that prefix are not exhaustively reconsidered.

If several candidates have the same largest amplitude, one is selected at random. Equal-amplitude states can have different phases and different subsequent continuations. The revised scheme can recover some alternatives excluded by the native update, but it does not retain every history; tie-breaking can, therefore, affect its final result.

For a shared prefix $S\left(p\right)=S^{o}\left(p\right)$, a possible delayed reversal of the amplitude ordering is

(19) $$\begin{array}{c} \left\{\begin{array}{c} \left|S(p+1)\right|\geq \left|S^{o}\left(p+1\right)\right|\\ \left|S(p+2)\right|\geq \left|S^{o}\left(p+2\right)\right|\\ \left|S(p+3)\right|<\left|S^{o}\left(p+3\right)\right| \end{array}\right.. \end{array}$$

These inequalities illustrate how an earlier discarded trajectory can become preferable after the limited comparison window has passed. They neither characterize all possible failures nor establish a general approximation ratio.

**Figure 5:**
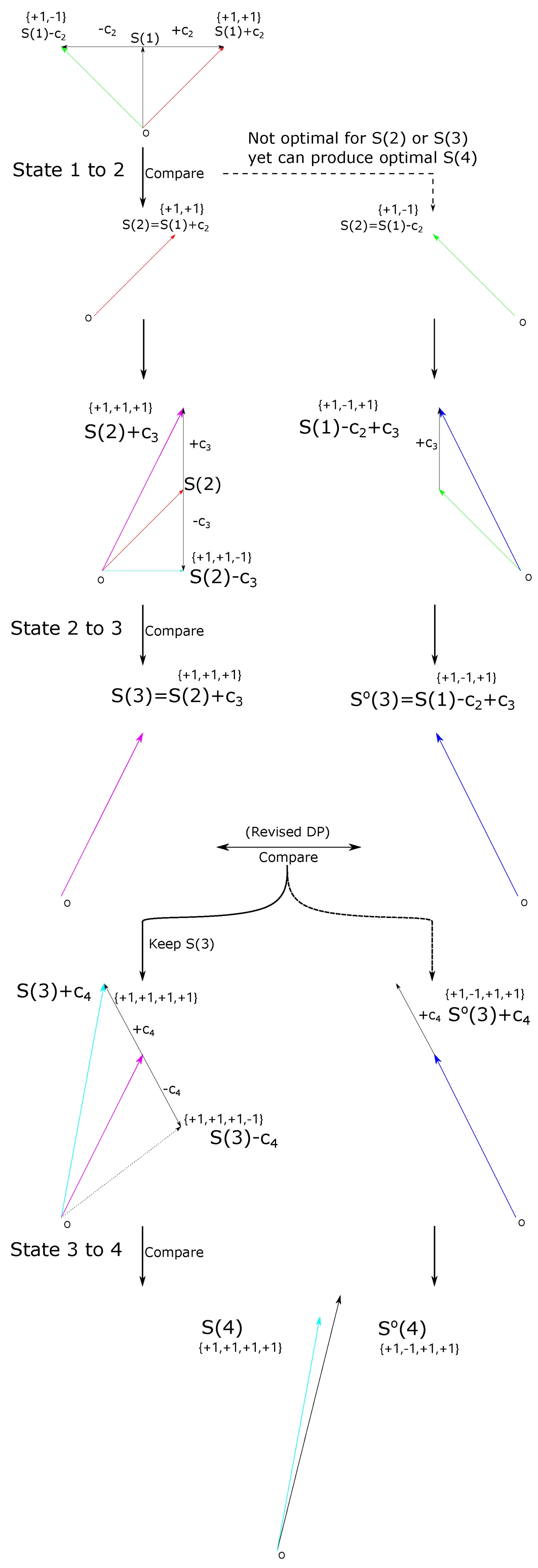
An example of sub-optimality of the revised DP.

To demonstrate that suboptimality remains possible for the revised scheme, consider the four-coefficient example in Figure 5. Let $r_{i}=\alpha_{i}e^{j\gamma_{i}}$, with $\alpha_{1}=\alpha_{2}=\alpha_{3}=\alpha_{4}=1$ and phases $\gamma_{1}=0$, $\gamma_{2}=\pi /2$, $\gamma_{3}=0$, and $\gamma_{4}=-\arctan\left(1/2\right)$, respectively. The equal magnitudes allow this ordering. At stage 3, the candidates 2+j and 2−j have equal amplitude. Suppose tie-breaking retains S(3)=2+j. At stage 4, the restricted update can reconsider the signs of $r_{3}$ and $r_{4}$, but does not revisit the discarded sign of $r_{2}$. The retained and globally maximizing trajectories are

(20) $$\begin{array}{cc} \; & S\left(1\right)=S^{o}\left(1\right)=1,\\ & S\left(2\right)=1+j,\;S^{o}\left(2\right)=1-j\\ & S\left(3\right)=1+e^{j\pi /2}+1=2+j\\ & S^{o}\left(3\right)=1-e^{j\pi /2}+1=2-j. \end{array}$$

The selected S(4) derived from S(3) and the actual optimal $S^{o}\left(4\right)$ derived from $S^{o}\left(3\right)$ are

(21) $$\begin{array}{cc} \; & S\left(4\right)=\left(2+\frac{2}{\sqrt{5}}\right)+j\left(1-\frac{1}{\sqrt{5}}\right) \end{array}$$

and

(22) $$\begin{array}{c} S^{o}\left(4\right)=\left(2+\frac{2}{\sqrt{5}}\right)-j\left(1+\frac{1}{\sqrt{5}}\right) \end{array}$$

respectively. For this coefficient sequence, the largest possible amplitude after the first three coefficients is $\sqrt{5}$. The triangle inequality, therefore, bounds the final amplitude by $\sqrt{5}+1$, which is attained by $S^{o}\left(4\right)$ because $r_{4}$ is aligned with 2−j. Hence,

$$\left|S\left(4\right)\right|=\sqrt{6+6/\sqrt{5}}\approx 2.9467,\;\left|S^{o}\left(4\right)\right|=\sqrt{5}+1\approx 3.2361.$$

The amplitude ratio is approximately 91.06%, and the corresponding squared-amplitude (local beamforming-gain) ratio is approximately 82.92%. These are case-specific values for the stated tie-breaking outcome. Selecting 2−j at stage 3 attains the optimum in this example. Neither outcome establishes a universal performance bound.

More generally, an amplitude-ordering reversal may occur beyond the comparison window. One illustrative pattern, with x>2, is

(23) $$\begin{array}{c} \left\{\begin{array}{c} \left|S(p+1)\right|\geq \left|S^{o}\left(p+1\right)\right|\\ \left|S(p+2)\right|\geq \left|S^{o}\left(p+2\right)\right|\\ \ldots \\ \left|S(p+x)\right|<\left|S^{o}\left(p+x\right)\right| \end{array}\right., \end{array}$$

where the intermediate inequalities describe the retained trajectory before the reversal. This schematic pattern does not establish how frequently reversals occur or bound their eventual loss. The finite candidate comparisons and antenna ordering, therefore, support a low-complexity heuristic, while the reported simulations assess its performance under the investigated settings.

**Figure 6:**
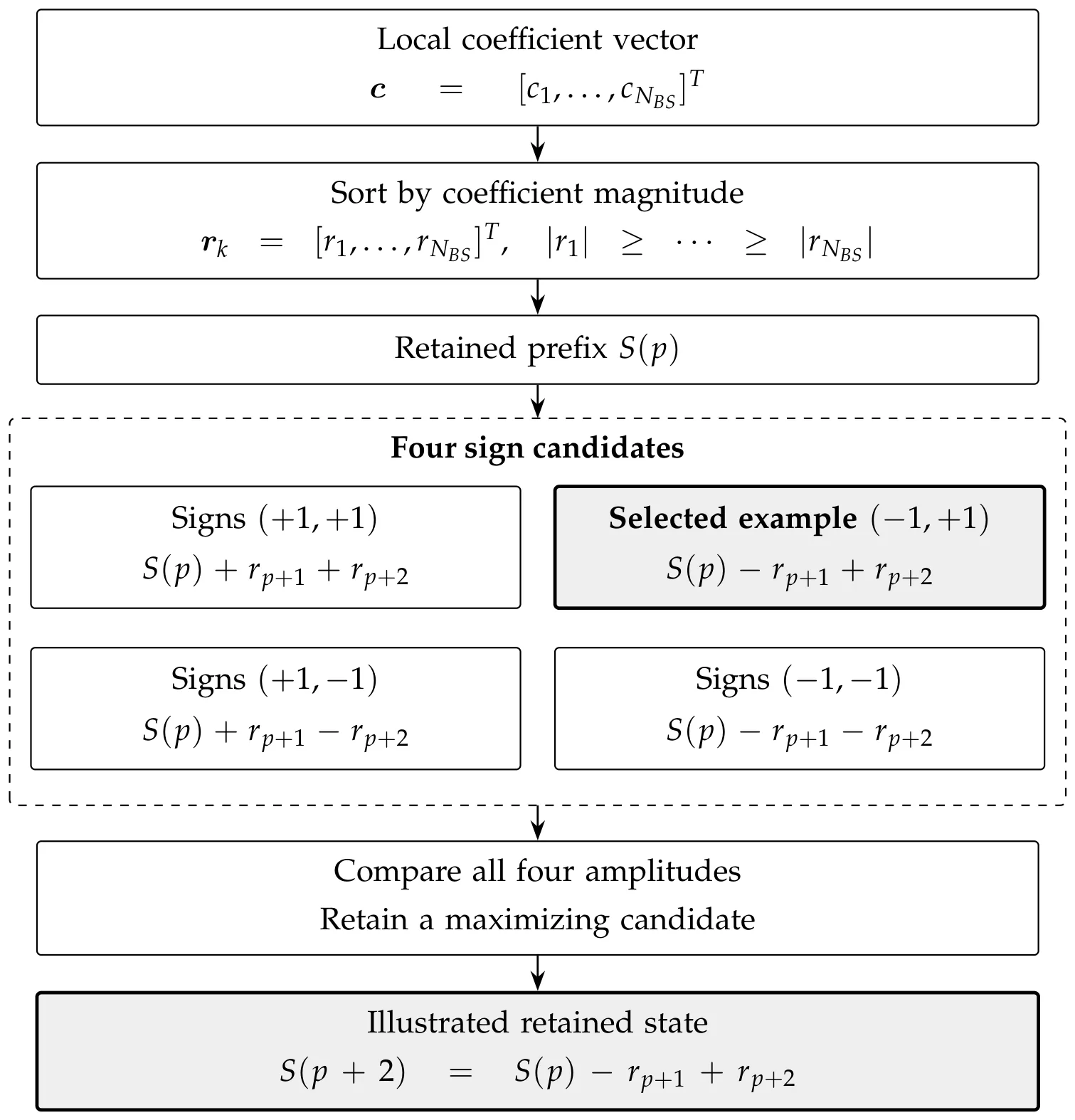
The revised DP update using the sorted local coefficient vector $r_{k}$. Here $c_{q}=\alpha_{q}e^{j\gamma_{q}}$, $\alpha_{q}=\left|c_{q}\right|$, and $r_{j}=c_{b_{j}}$, where $b_{j}$ is the physical antenna index at sorted position *j*. For a retained prefix S(p), the four candidates correspond to the sign pairs for $r_{p+1}$ and $r_{p+2}$. The bold border and “Selected example” label identify the illustrated retained candidate; its first sign is revised to −1.

A block diagram of the revised DP is shown in Figure 6. Its input is the local coefficient vector c, and $r_{k}$ is the vector after amplitude ordering. For a fixed prefix S(p), both $S\left(p\right)+r_{p+1}$ and $S\left(p\right)-r_{p+1}$ are considered when the next sorted coefficient $r_{p+2}$ is processed. In the illustrated outcome, the candidate $S\left(p\right)-r_{p+1}+r_{p+2}$ is retained, and the sign associated with $r_{p+1}$ is revised. A bold border and the label “Selected example” identify this candidate. Sorted position p+1 corresponds to physical antenna $b_{p+1}$. This depicts a correction within the retained candidate set, not a guarantee of a globally optimal final sequence.

### 4.5. Applicability to Wideband Channels

To clarify the scope of the formulation, consider an OFDM system with $N_{\mathrm{sc}}$ subcarriers indexed by ν. The shared-analog and subcarrier-dependent digital framework follows wideband hybrid processing [15] (Section II-A). We apply this framework to an ideal SI network whose response is assumed frequency-independent over the occupied band, with a common analog precoder and antenna-activation pattern. In particular,

$$H_{e}\left[\nu \right]=\left[\begin{array}{c} h_{1}{\left[\nu \right]}^{H}F_{RF}\\ \vdots \\ h_{K}{\left[\nu \right]}^{H}F_{RF} \end{array}\right],\;F_{RF}\left[\nu \right]=F_{RF}.$$

Applying the ZF pseudoinverse defined in Section 3 separately to each full-row-rank $H_{e}\left[\nu \right]$, with an illustrative common scaling on each subcarrier, gives

$$F_{BB}\left[\nu \right]=\eta_{\nu }H_{e}{\left[\nu \right]}^{H}{\left(H_{e}\left[\nu \right]H_{e}{\left[\nu \right]}^{H}\right)}^{-1},$$

where the factors $\eta_{\nu }$ implement the chosen power allocation and normalization so that the subcarriers collectively satisfy the total transmit-power budget. Thus, subcarrier-dependent digital processing is compatible with a common SI analog network. The single digital matrix in the present formulation corresponds to the frequency-flat model.

Within that wideband framework, we derive a sufficient condition for consistent local SI beamforming decisions under an additional proportional-channel assumption. Let $h_{k,i}\left[\nu \right]$ denote the restriction of user *k*’s channel to the fixed subarray connected to RF chain *i*, and let $f_{i}$ be its SI weight vector. Suppose that the spatial channel vectors are proportional across subcarriers:

$$h_{k,i}\left[\nu \right]=a_{k,i}\left[\nu \right]h_{k,i,0}.$$

Substituting this assumption into the local squared-magnitude gain gives, for any feasible $f_{i}$,

$$G_{k,i,\nu }\left(f_{i}\right)={\left|h_{k,i}{\left[\nu \right]}^{H}f_{i}\right|}^{2}={\left|a_{k,i}\left[\nu \right]\right|}^{2}{\left|h_{k,i,0}^{H}f_{i}\right|}^{2}.$$

For fixed nonnegative weights $w_{\nu }$ with $C_{k,i}>0$, a weighted aggregate of these gains is, therefore,

$$\begin{array}{cc} {\bar{G}}_{k,i}\left(f_{i}\right) & =\sum_{\nu =1}^{N_{\mathrm{sc}}}w_{\nu }G_{k,i,\nu }\left(f_{i}\right)=C_{k,i}{\left|h_{k,i,0}^{H}f_{i}\right|}^{2},\\ C_{k,i} & =\sum_{\nu =1}^{N_{\mathrm{sc}}}w_{\nu }{\left|a_{k,i}\left[\nu \right]\right|}^{2}. \end{array}$$

Multiplication by the positive factor $C_{k,i}$ preserves the ordering of all feasible local analog configurations. With a fixed antenna ordering and consistent tie-breaking, the amplitude comparisons used in the DP updates likewise preserve their ordering under this scaling. A single propagation path with a frequency-independent array response is one example of this proportional spatial structure. This conditional result concerns the local analog gain and does not establish optimality of the DP scheme or transfer its rate and EE results to a general wideband multi-user system.

For general multipath channels, distinct path delays and frequency-dependent array responses can change the spatial channel direction across subcarriers. A joint extension would need to maintain subcarrier-dependent states with a shared antenna decision, for example

$$S_{\nu }\left(p+1\right)=S_{\nu }\left(p\right)+\delta \left(p+1\right)c_{p+1,\nu },$$

where $c_{p+1,\nu }$ is the channel coefficient of the next antenna on subcarrier ν, and the same δ(p+1) is used on every subcarrier. The decision set is {−1,1} when all antennas are active and includes 0 when deactivation is allowed. Antenna ordering, candidate evaluation, and QoS constraints would have to account for all subcarriers. The proportional-channel identity above does not determine the wideband sum-rate or EE objective, which also depends on digital processing and power allocation. Consequently, the algorithm analysis and numerical performance reported here apply to the frequency-flat model; the wideband discussion establishes the architecture requirements and the stated conditional gain relationship.

## 5. EE-Oriented DP Precoding with Antenna Deactivation

The EE metric in this section remains $\xi =R/P_{all}$ as defined in Section 3. Antenna-count minimization is the circuit-power surrogate used to construct candidate configurations; their EE is evaluated from the resulting sum rate and total power. Assuming time-division duplex (TDD) operation and channel reciprocity, the estimated uplink channel is used for downlink precoding within the channel coherence time. Switching off antennas can reduce circuit power, but can also change the achievable rate and the inverter configuration. We, therefore, seek configurations that reduce the active-antenna count while preserving a prescribed beamforming-gain requirement. This is an EE-oriented surrogate design; its relationship to the rate-to-power objective is analyzed below. The antenna states are extended to δ(p)∈{−1,0,1} for $p=1,\ldots ,N_{BS}$.

### 5.1. Precoding Scheme with Antenna Elimination

For antenna deactivation, we reconstruct the prefix sums of the selected antenna-state configuration and use their amplitude increments to order candidate removals. These increments are low-complexity contribution estimates, rather than exact sum-rate contributions or exact final gain losses after removing an antenna and updating the precoder. Using this ordering, we seek to deactivate antennas while satisfying a prescribed quality-of-service (QoS) surrogate expressed in terms of analog beamforming gain. The resulting antenna-count minimization problem is

(24) $$\begin{array}{cc} \delta \left(1\right),\cdots ,\delta \left(N_{BS}\right) & =\underset{\delta \left(1\right)\cdots \delta \left(N_{BS}\right)}{\arg\min}\sum_{p=1}^{N_{BS}}\left|\delta \left(p\right)\right|,\\ s.t.\;{∥h_{k}^{H}F_{RF}∥}_{2}^{2} & \geq \Omega_{k},\;k=1,\ldots ,K\\ \delta \left(p\right) & \in \left\{-1,0,1\right\},p=1\cdots N_{BS}, \end{array}$$

where $\Omega_{k}$ is the prescribed analog beamforming-gain threshold for MS *k*. Write $G_{k}={∥h_{k}^{H}F_{RF}∥}_{2}^{2}$. The objective in (24) is the active-antenna count, since |δ(p)| equals one for either active sign and zero for an inactive antenna. Thus, (24) defines a gain-constrained antenna-count problem, not a new definition of EE or a direct maximization of $R/P_{all}$. We assume that the initial all-active configuration satisfies these gain constraints. A DP-based sequential approach is used to seek feasible configurations with fewer active antennas; it does not guarantee a globally minimum active-antenna count.

*Relationship to the EE objective.* The following rate–power relationships are derived for the antenna-count surrogate proposed in (24), using the EE definition and SI power accounting in Section 3. Let *x* denote a feasible antenna-state configuration together with its associated digital precoder, and let F collect such configurations satisfying the gain constraints under the same channel, RF-chain count, and total transmit-power setting. The EE objective over this set is $\xi^{★}=\max_{x\in F}R\left(x\right)/P\left(x\right)$, where $P\left(x\right)=P_{all}\left(x\right)$ follows (7). Problem (24) minimizes a different objective. For fixed $P_{BB}$, $N_{RF}$, and ρ, the power model becomes

$$P\left(x\right)=P_{0}+N_{BS}^{A}\left(x\right)P_{SW}+N_{BS}^{I}\left(x\right)P_{IN},\;P_{0}=P_{BB}+N_{RF}P_{RF}+\rho .$$

Consequently, active-antenna count is a proxy for circuit power, but does not fully determine it when the inverter count also changes.

Consider any feasible deactivation step with rate and power changing from R,P to $R^{\prime }=R-\Delta R$ and $P^{\prime }=P-\Delta P$, where R>0, P>0, and $P^{\prime }>0$. Direct comparison gives

$$\frac{R-\Delta R}{P-\Delta P}\geq \frac{R}{P}\;⟺\;\frac{\Delta R}{R}\leq \frac{\Delta P}{P}.$$

Thus, EE improves strictly when the relative rate loss is smaller than the relative net power saving. For one deactivated antenna with the fixed parameters above,

$$\Delta P=P_{SW}+\left(N_{BS}^{I}-N_{BS}^{I\prime }\right)P_{IN},$$

where $N_{BS}^{I\prime }$ is the inverter count after deactivation and all associated sign updates. If the remaining signs do not change, the saved power is $P_{SW}$ or $P_{SW}+P_{IN}$ depending on the removed antenna’s state. With sign updates, the actual net saving must include changes at the other antennas. Likewise, ΔR must use the resulting sum rate in (6), including the digital-precoding and normalization effects. The gain-based ordering seeks to limit performance loss, but neither it nor the QoS constraint alone enforces this EE-improvement condition. Even if the condition holds at every accepted step, it establishes monotonic improvement along that path, not global optimality.

A sufficient condition for exact equivalence is that every compared feasible configuration has the same rate $R_{0}>0$, while its power depends only on active-antenna count and is strictly increasing, for example $P\left(x\right)=P_{0}+p_{a}N_{BS}^{A}\left(x\right)$ with $p_{a}>0$. A globally minimum-cardinality solution then maximizes EE over the same feasible set. Equal achievable rates and such a power-count relationship are additional assumptions, rather than consequences of the gain constraint.

A bound derived from the same EE definition further describes the role of rate variation and power reduction under additional assumptions. Suppose that $0<R_{\min}\leq R\left(x\right)\leq R_{\max}$ for every x∈F, and a feasible solution $x_{s}$ satisfies $P\left(x_{s}\right)\leq \left(1+\eta \right)P_{\min}$, where $P_{\min}=\min_{x\in F}P\left(x\right)>0$ and η≥0. Since

$$\xi \left(x_{s}\right)\geq \frac{R_{\min}}{\left(1+\eta \right)P_{\min}},\;\xi^{★}\leq \frac{R_{\max}}{P_{\min}},$$

it follows that

$$\frac{\xi (x_{s})}{\xi^{★}}\geq \frac{R_{\min}}{\left(1+\eta \right)R_{\max}}\geq \frac{1}{\left(1+\eta )(1+\varepsilon \right)}\;\mathrm{if}\;R_{\max}\leq \left(1+\varepsilon \right)R_{\min},\;\varepsilon \geq 0.$$

This bound requires both small rate variation and proximity to minimum total power. Neither η nor ε is bounded for the proposed sequential method, and minimizing antenna count does not by itself establish the required power bound. Thus, this is a conditional relationship, not an approximation guarantee for the algorithm.

Finally, $G_{k}\geq \Omega_{k}$ is an analog-gain requirement, not a direct constraint on the post-ZF SINR or sum rate. The effective-channel geometry and digital-precoder normalization also affect these quantities. Retaining a fraction of the analog gain, therefore, does not generally imply retaining the same fraction of the achievable rate. Throughout the evaluation, EE refers to the resulting rate divided by the full power in (7); the deactivation rule uses the gain constraint in (24), without an additional EE-monotonicity acceptance test.

To define the contribution estimates, consider one fixed subarray of length $n=n_{i}$ and retain the same local user/subarray design target as in the all-active stage. Let p=1,…,n denote a sorted position, $b_{p}\in A_{i}$ the physical antenna at that position, and $r_{p}=c_{b_{p}}$ its local coefficient, with $|r_{1}\left|\geq \cdots \geq \right|r_{n}|$. Zero coefficients outside this subarray are omitted. The all-active signs are obtained by the revised DP in Section 4.4. After its sign revisions are complete, reconstruct the prefixes of the resulting configuration as

$$S_{0}=0,\;S_{p}=\sum_{j=1}^{p}\delta \left(b_{j}\right)r_{j},\;p=1,\ldots ,n.$$

Here $S_{p}$ is indexed by sorted position, whereas $\delta (b_{p})$ is indexed by physical antenna. Define one contribution estimate for every antenna, including the first:

(25) $$\begin{array}{cc} t & ={\left[t_{1},\ldots ,t_{n}\right]}^{T},\\ t_{p} & =|S_{p}\left|-\right|S_{p-1}|,\;p=1,\ldots ,n. \end{array}$$

The first estimate is $t_{1}=\left|S_{1}\right|$. The other estimates are signed increments of the reconstructed prefixes. They need not be nonnegative, because the revised look-ahead procedure can change an earlier sign; its final configuration need not maximize each truncated prefix. These estimates provide a heuristic removal priority, rather than exact marginal gain or rate losses. Sort the original value–antenna pairs using a permutation π:

(26) $$\begin{array}{cc} t^{\prime } & ={\left[t_{\pi (1)},\ldots ,t_{\pi (n)}\right]}^{T},\\ d_{j} & =b_{\pi (j)},\;t_{\pi (1)}\leq \cdots \leq t_{\pi (n)}. \end{array}$$

The permutation changes the removal priority, not the original prefix order; no new differences between adjacent ranked prefixes are taken. Apply this construction separately within each fixed subarray and concatenate the tagged estimates before the array-wide ranking. The resulting physical-antenna list is denoted by $d_{1},\ldots ,d_{N_{BS}}$, with subarray membership and original sorted positions retained. Local sums are never combined coherently across different RF chains. The ranking is formed from the initial all-active configuration and is kept fixed during this removal pass.

Beginning with $d_{1}$, copy the accepted state vector into a trial vector and tentatively set the selected antenna to zero. Rebuild the trial analog precoder after any local sign updates, and evaluate ${\tilde{G}}_{k}={∥h_{k}^{H}{\tilde{F}}_{RF}∥}_{2}^{2}$ for every MS. The trial is accepted only if ${\tilde{G}}_{k}\geq \Omega_{k}$ for all *k*. Otherwise, discard the entire trial, retain the preceding accepted configuration, and terminate the removal pass. If the all-active initialization does not satisfy the thresholds, the procedure reports an initialization failure rather than declaring the optimization problem infeasible. The actual sum rate and EE are evaluated separately using (5)–(7), with the associated digital precoder under the rank condition in Section 3. No additional EE-monotonicity test is imposed.

### 5.2. Precoding Scheme with Revised DP

Deactivating an antenna changes the retained partial sums and can change the preferred signs of subsequent antennas. We, therefore, introduce a DP-based deactivation approach that re-evaluates affected sign decisions to mitigate the resulting beamforming loss. This procedure remains a sequential heuristic and does not guarantee a globally optimal joint choice of antenna activation and signs.

Lemma 1 motivates examining both the magnitude $|r_{p}|$ and the relative phase of a coefficient and its preceding sum. For a given local state, a small coefficient or a nearly perpendicular contribution can have a limited amplitude effect. This is a local mechanism, rather than an exact characterization of the final multi-user gain loss. If the trial removes physical antenna $q=b_{a}$ at sorted position *a*, its contribution vanishes and ${\tilde{S}}_{a}=S_{a-1}$. Later positions are p>a in this same subarray; they are not the physical antenna numbers q+1,q+2,….

Using the squared-magnitude expansion in Section 4.3, the local two-sign maximizer for an active coefficient $r_{p}$ and a fixed preceding state, and the resulting phase, can be written as

(27) δ(bp)∈argmaxδ∈{−1,1}δRe{Sp−1*rp},Sp=Sp−1+δ(bp)rp,γSp=arg{Sp},Sp≠0.

The maximization in (27) is a conditional local comparison, not a property of every sign in the final revised-DP configuration. The state identity remains valid for any selected sign; the acute-angle analysis below additionally assumes the stated two-sign maximizer. The argument function handles all quadrants of a nonzero sum. If the real-part comparison is zero, both signs give the same amplitude and the existing active sign may be retained. A zero sum has no defined phase and is handled by direct amplitude comparison.

For $S_{p-1}\neq 0$ and $r_{p}\neq 0$, the local maximizing sign makes Re{Sp−1*δ(bp)rp}≥0. Rotate the complex plane so that the preceding sum lies on the positive real axis. Let $\gamma^{-}\in \left[0,\pi /2\right]$ be the nonnegative angle between that sum and the selected signed coefficient. The magnitude of the angular change is

(28) $$\Delta \gamma_{p}=\arctan\left(\frac{|r_{p}|\sin\gamma^{-}}{|S_{p-1}\left|+\right|r_{p}|\cos\gamma^{-}}\right).$$

Here $|S_{p-1}|>0$ makes the denominator positive. The maximum over this angle interval is $\arctan(|r_{p}|/|S_{p-1}|)$ at $\gamma^{-}=\pi /2$. A zero coefficient has zero angular impact; a zero preceding sum has no reference direction, so the candidate comparison is used directly.

**Figure 7:**
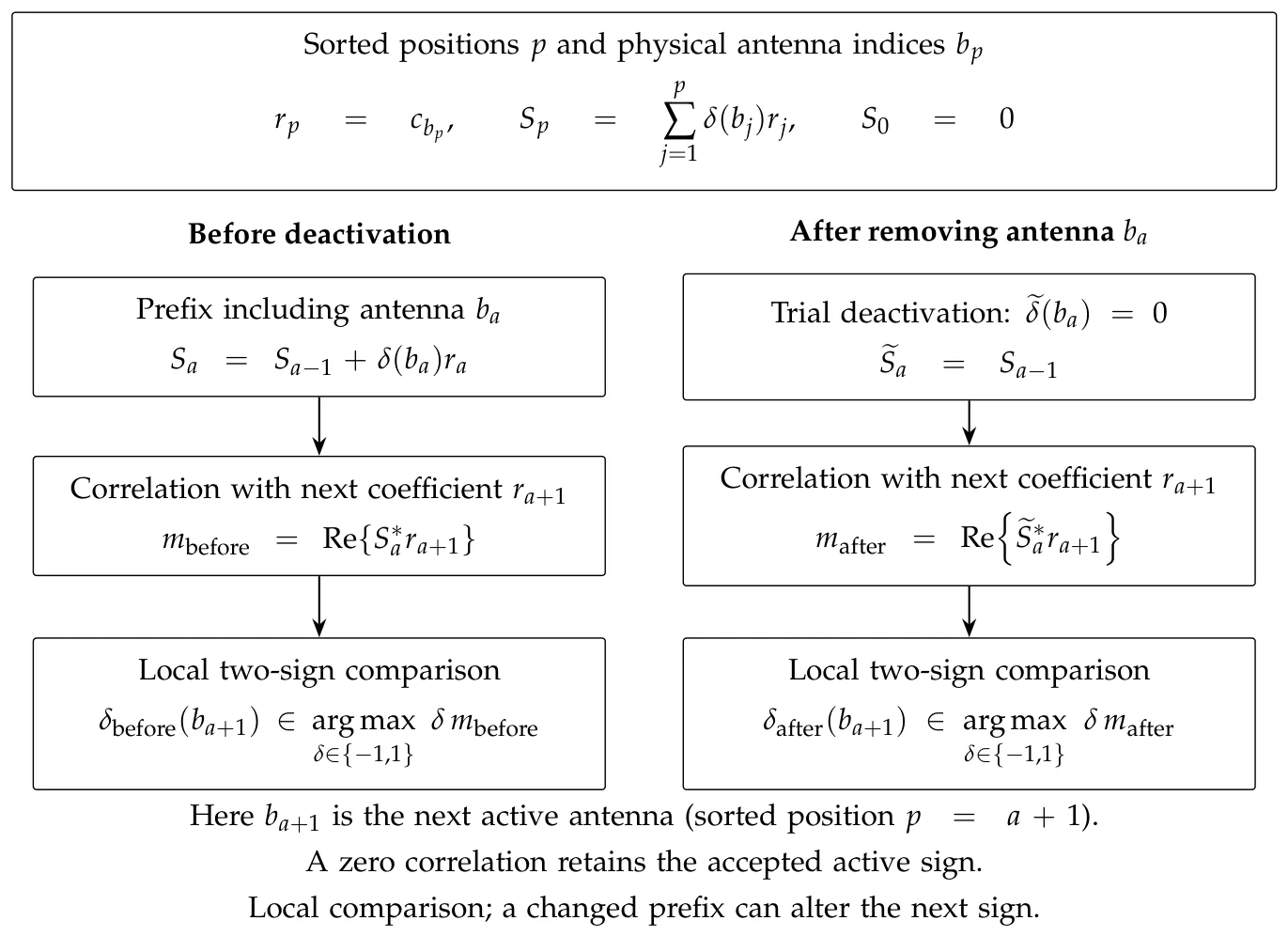
Local sign comparison before and after antenna $b_{a}$ is deactivated. The index *a* denotes a sorted position and $b_{a}$ its physical antenna. A changed prefix can alter the preferred sign of the next active coefficient; the illustration does not assert global optimality.

This angular change describes the difference between the directions of the preceding and updated partial sums after the sign has been selected. Removing the antenna can consequently alter later sign decisions. Figure 7 illustrates such a change for a fixed preceding state, rather than a global optimality claim.

Expression (28) describes the angular mechanism under its local two-sign and nonzero-state conditions. During deactivation, the revised DP supplies the all-active initialization, while subsequent local sign repair uses the two-sign comparison at the updated prefix. For a later active position p>a, set mp=Re{S˜p−1*rp}. The trial sign is

(29) $$\tilde{\delta }\left(b_{p}\right)=\left\{\begin{array}{cc} 1, & m_{p}>0,\\ -1, & m_{p}<0,\\ \delta (b_{p}), & m_{p}=0. \end{array}\right.$$

The tie retains the accepted sign of that same physical antenna. Update ${\tilde{S}}_{p}={\tilde{S}}_{p-1}+\tilde{\delta }\left(b_{p}\right)r_{p}$. Already deactivated antennas retain sign zero and are skipped by the active-sign comparison, so a repair does not reactivate them.

Algorithm 1 distinguishes accepted and trial configurations. To limit sign-repair work, it retains the heuristic of stopping when an active trial sign equals its accepted value. A sign equality does not imply equality of the complex prefixes or of all later preferred signs. The unvisited signs are, therefore, retained as a heuristic choice, not as an exact propagation result. After stopping, reconstruct every trial prefix from the complete trial sign vector and evaluate all-user gain using the rebuilt analog matrix. No stale stored state is used for feasibility. Thus, the gain constraints govern acceptance even when sign repair is truncated, without providing a global optimality or EE-monotonicity guarantee.
**Algorithm 1** DP-Based SI Analog Precoder Design with Antenna Deactivation**Input:** Channel information, fixed subarray design targets, thresholds $\Omega_{k}$**Output:** Last accepted gain-feasible antenna-state vector δ  1:Obtain all-active δ using Section 4.4 within each subarray.  2:**if** some $G_{k}<\Omega_{k}$ **then**  3:   Report initialization failure and stop.  4:**end if**  5:Reconstruct each local prefix and all $n_{i}$ estimates using (25).  6:Sort the tagged estimates from all subarrays as in (26); obtain $d_{1},\ldots ,d_{N_{BS}}$.  7:**for** j=1 **to** $N_{BS}$ **do**  8:   Set trial $\tilde{\delta }=\delta$ and $q=d_{j}$; identify its subarray and sorted position *a*.  9:   Set $\tilde{\delta }\left(q\right)=0$ and ${\tilde{S}}_{a}=S_{a-1}$ in that subarray.10:   **for** p=a+1 **to** $n_{i}$ **do**11:     **if** $\tilde{\delta }\left(b_{p}\right)\neq 0$ **then**12:        Apply (29) and update ${\tilde{S}}_{p}$.13:        **if** $\tilde{\delta }\left(b_{p}\right)=\delta \left(b_{p}\right)$ **then**14:          **break** (heuristic sign-repair stop).15:        **end if**16:     **else**17:        Set ${\tilde{S}}_{p}={\tilde{S}}_{p-1}$; retain the zero sign.18:     **end if**19:   **end for**20:   Reconstruct all trial prefixes and build ${\tilde{F}}_{RF}$ from $\tilde{\delta }$.21:   Compute ${\tilde{G}}_{k}={∥h_{k}^{H}{\tilde{F}}_{RF}∥}_{2}^{2}$ for all *k*.22:   **if** ${\tilde{G}}_{k}\geq \Omega_{k}$ for every *k* **then**23:     Accept $\delta =\tilde{\delta }$ and its reconstructed states.24:   **else**25:     Discard the trial; **break** with δ unchanged.26:   **end if**27:**end for**28:**Output:** The last accepted vector δ.

### 5.3. Complexity Analysis

We distinguish local candidate comparisons, complete system-objective evaluations, and measured execution time. These are different measures of computational cost. The proposed method reuses a retained complex partial sum: the native update compares two signs, and the revised update compares four sign combinations conditioned on a retained prefix. Each candidate uses a constant number of scalar complex operations. The cross-entropy method generates complete analog candidates, evaluates their performance, and updates the sampling distribution [22]; the coordinate-update method tests alternative states for one antenna while holding the others fixed and repeats the process over successive scans [23]. Table 2 summarizes these mechanisms.

For a local subarray sequence of length $n_{i}$, amplitude ordering costs $O(n_{i}\log n_{i})$ on average with quicksort, and the revised sequential comparisons cost $O(n_{i})$. A conservative bound is four local amplitude evaluations per update, or at most $4(n_{i}-1)$ for one pass. If each block is processed once, summing over the fixed subarrays gives $O(\sum_{i}n_{i}\log n_{i}+N_{BS})$, bounded by $O(N_{BS}\log N_{BS}+N_{BS})$. Additional local passes for different design targets must be counted separately. The ordering is motivated by processing larger coefficients first; it does not remove the remaining suboptimality analyzed in Section 4.3 and Section 4.4.

Antenna deactivation incurs contribution ranking and sign repair beyond the all-active construction. Let *T* be the number of attempted deactivations, including a final rejected trial if present, and let $\ell_{j}$ be the number of local positions visited during sign repair in trial *j*. Define

$$L=\sum_{j=1}^{T}\ell_{j}.$$

For one array-wide pass and fixed candidate counts per position, the scalar selection and ranking workload is $O(N_{BS}\log N_{BS}+L)$. In the conservative case $\ell_{j}\leq N_{BS}$ and $T\leq N_{BS}$, this component is bounded by $O(N_{BS}\log N_{BS}+N_{BS}^{2})$. The heuristic stop can reduce *L*, but every trial still reconstructs the complete prefixes, rebuilds the analog matrix, and checks all-user gain. Let $C_{G}$ include these per-trial reconstruction and feasibility costs; in particular, prefix reconstruction contributes $O(N_{BS})$ even after an early stop. With *Q* complete rate/EE evaluations of cost $C_{E}$, a workload accounting is

$$C_{\mathrm{DP}}=O\left(N_{BS}\log N_{BS}+L\right)+O\left(TC_{G}+QC_{E}\right).$$

This expression separates local state reuse from system-level checks; it is not a measured FLOP count or runtime.

For the cross-entropy budget I=20 and S=200, the principal candidate-evaluation count is IS=4000. The reference analysis reports dominant arithmetic scaling $O(ISN_{BS}K^{2})$ for its effective-channel and digital-processing calculations [22]. Coordinate-update complexity is counted in objective evaluations [23]: testing *q* states for every one of $N_{BS}$ coordinates over $t_{\max}$ complete scans gives $qN_{BS}t_{\max}$ principal candidate evaluations. With the two SI sign states {−1,1}, q=2; allowing the additional deactivation state 0 gives q=3. For $N_{BS}=128$ and a budget of $t_{\max}=30$ complete scans, these counts are 7680 and 11,520, respectively. They exclude initialization and other bookkeeping, and early stopping or reuse of an unchanged candidate can reduce the evaluations. The CE population size *S* is not a coordinate-update population parameter.

Table 3 fixes $N_{BS}=128$, $K=N_{RF}=4$, and $n_{i}=32$. For one all-active pass per fixed subarray, the array-wide local amplitude count $E_{A}$ satisfies

$$E_{A}\leq \sum_{i=1}^{N_{RF}}4\left(n_{i}-1\right)=4\left(N_{BS}-N_{RF}\right)=496,\;\frac{E_{A}}{N_{BS}}\leq 4\left(1-\frac{N_{RF}}{N_{BS}}\right)=3.875.$$

Repeated design passes are additional. For equal-size blocks, average sorting work per installed antenna scales as $O(\log\left(N_{BS}/N_{RF}\right))$. Normalizing the deactivation cost model by the common installed count $N_{BS}$, rather than the selected active count, gives

$$\frac{C_{\mathrm{DP}}}{N_{BS}}=O\left(\log N_{BS}+\frac{L}{N_{BS}}\right)+O\left(\frac{TC_{G}+QC_{E}}{N_{BS}}\right).$$

The cost $C_{G}$ retains full trial reconstruction. CE has dominant normalized arithmetic scaling $O(ISK^{2})$; its 4000 candidate evaluations each cover the complete array. CU has $qt_{\max}$ coordinate-candidate objective evaluations per antenna, whose individual costs remain necessary. These normalized measures retain their distinct units.

The common array size and the per-antenna normalization make the processing scope and antenna-size dependence explicit. One local amplitude evaluation does not have the same cost as a system-objective evaluation involving effective-channel and digital-precoder computations, and normalization does not make these units equivalent. Moreover, matrix updates, caching, and parallelization can change the implementation cost of the benchmarks. We, therefore, do not divide the counts in Table 3 to infer runtime speedups. The budgets describe the stated search settings rather than equal time budgets or equal accuracy targets. Execution time also depends on the hardware, numerical libraries, and implementation. The supported mechanism for reduced search workload is partial-sum reuse and limited local candidate evaluation, with the performance trade-off assessed under the reported settings in Section 6.

## 6. Simulation Results

All results in this section use the frequency-flat narrowband channel model described in Section 3 and the numerical setting $N_{RF}=K$. When $N_{RF}$ is swept, *K* is varied together with it. This uses the minimum RF-chain count compatible with the specified stream count under the full-row-rank condition; it is not asserted to maximize EE or rate over all possible RF-chain counts. These results do not isolate the effect of increasing $N_{RF}$ at a fixed *K*, and do not provide numerical validation for $K<N_{RF}$.

The numerical study evaluates four aspects of the proposed hybrid precoding scheme. First, achievable multi-user sum rates are compared with all antennas active under the stated digital-processing and power-normalization rules. This evaluates the complete precoding chain, including the SI analog design in Section 4, rather than only its local analog beamforming gain. Second, the antenna-deactivation procedure in Section 5 is evaluated in terms of EE and compared with the stated benchmarks. Third, the behavior under different channel configurations and analog-gain thresholds is examined. Finally, sensitivity to channel estimation errors is evaluated. Simulation results are obtained under the following configurations, unless otherwise mentioned: The urban and rural labels denote simplified statistical propagation settings. In the simplified urban setting, each user’s path count $L_{k}$ is drawn uniformly from {1,2,3}; the simplified rural setting uses $L_{k}=1$. Each path is represented by one complex gain and one array steering vector in (3), without a separate hierarchy of clusters and rays within clusters. Both settings use the per-path gain and departure-angle distributions and the half-wavelength-spaced 16×8 UPA listed in Table 4. The polar and azimuth distributions specify the sampling ranges of path directions, not angular spreads about cluster centers; no cluster angular-spread parameter is assigned.

Here, CN(1,1) denotes a complex Gaussian path gain with mean 1 and variance 1; it is not a zero-mean Rayleigh-entry channel model. Equation (3) uses these coefficients without an additional path-count normalization factor. The comparison, therefore, examines different levels of spatial multipath richness under the stated per-path gain statistics, rather than isolating path count at equal total channel power. No distance-dependent path-loss law, path-loss exponent, or shadowing distribution is assigned. Carrier frequency, user distance, and site layout are also unspecified; performance is evaluated at prescribed SNR values. The urban/rural labels in the figures refer to these simplified settings, not calibrated coverage predictions for specific deployments.

The roles of the comparisons are distinguished explicitly. CE and CU are the SI search benchmarks, including the specified state-alphabet extension for antenna deactivation. Fully digital ZF, PS-based hybrid processing, and antenna selection serve as architecture or method references with different hardware freedoms. Table 1 additionally compares dynamic-subarray, wideband, and learning-assisted designs at the methodological level. Their external results are not substituted for controlled SI comparisons under the present channel and processing settings.

### 6.1. Achievable Sum-Rate Performance with All Antennas Active

The all-active precoding schemes are evaluated in terms of achievable multi-user sum rate. The numerical study focuses on the target $N_{BS}=128$ array to compare achievable performance and computational cost under common channel, hardware, and power settings. The adaptive cross-entropy [22] and coordinate-update [23] methods provide SI-architecture benchmarks.

For this 128-antenna configuration with all antennas active, direct enumeration of the $2^{128}$ binary SI sign patterns is computationally prohibitive. Here, exhaustive evaluation would mean identifying the best sum rate over feasible analog SI configurations under the prescribed ZF digital-precoding and power-normalization rules, including the full-row-rank requirement. This is distinct from jointly optimizing all analog and digital processing to determine a theoretical limit of the SI architecture. Exhaustive evaluation is feasible for sufficiently small arrays and provides a useful complementary assessment of finite-size optimality gaps. The comparisons reported here address performance at the target array size relative to the stated benchmarks, and do not quantify the gap to the globally best SI configuration under the prescribed processing rules.

The small analytical examples in Section 4.3 and Section 4.4 complement these comparisons by characterizing local analog-gain optima and illustrating how discarded intermediate states can lead to suboptimality. In particular, the four-coefficient example exhibits a suboptimal outcome for the specified tie-breaking choice. These examples concern a fixed user and subarray; they are not exhaustive-search evaluations of multi-user sum rate or EE. Their local amplitude ratios, therefore, cannot be interpreted as system-level optimality guarantees.

The fully digital ZF precoder and the alternating-optimization PS-based hybrid precoder [9] are included as performance references, together with the antenna-selection scheme [7]. These reference results are not treated as certified upper or lower bounds on the proposed algorithm.

**Figure 8:**
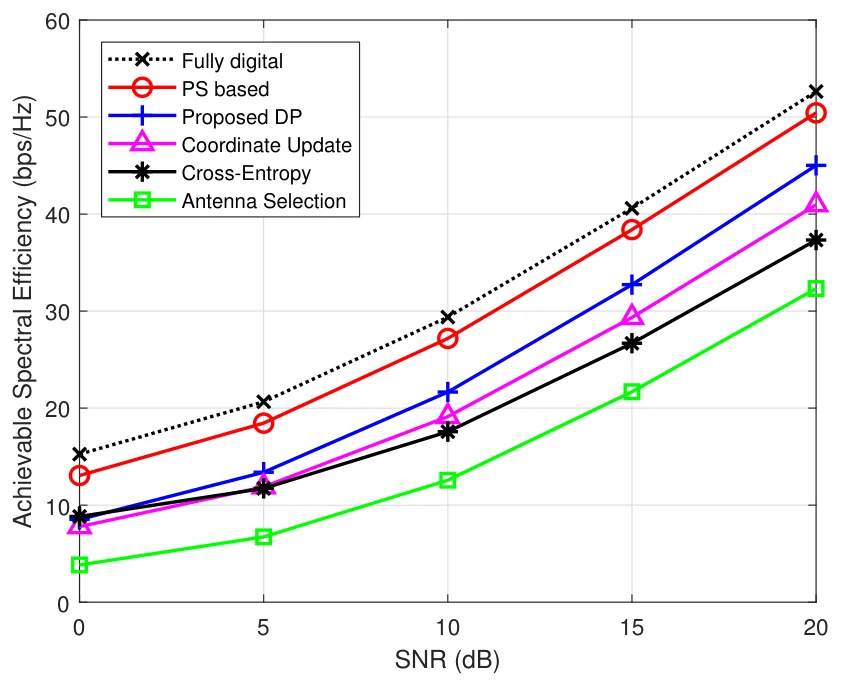
Achievable sum-rate comparison in the simplified urban setting ($L_{k}$ uniformly drawn from {1,2,3}); channel parameters are listed in Table 4.

**Figure 9:**
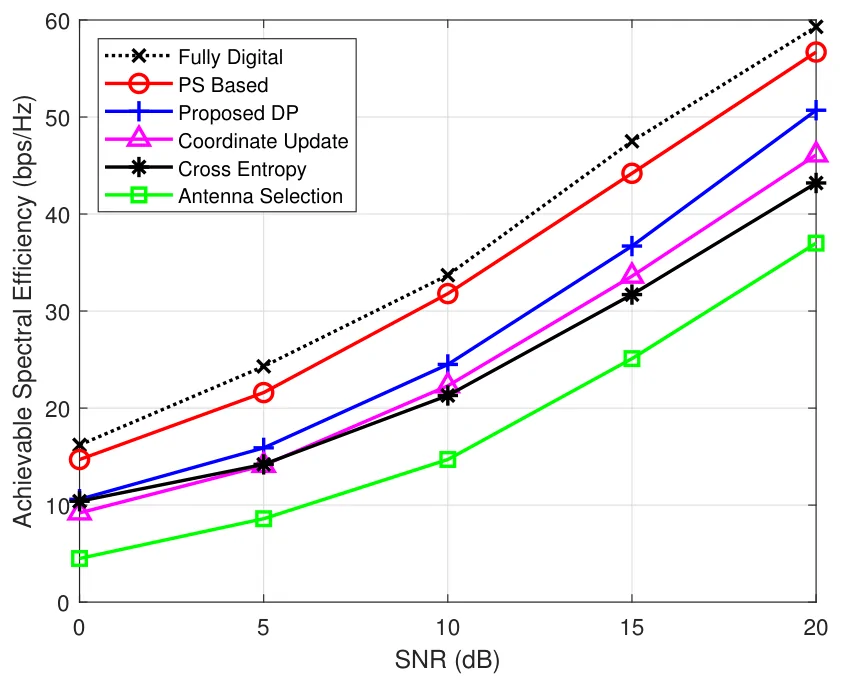
Achievable sum-rate comparison in the simplified rural setting ($L_{k}=1$); channel parameters are listed in Table 4.

Figure 8 and Figure 9 compare the all-active sum rates under the simplified urban multipath and simplified rural single-path settings in Table 4. To specify both reference methods explicitly, define

$$\Delta R_{b}=\frac{R_{\mathrm{DP}}-R_{b}}{R_{b}}\times 100\%,\;b\in \left\{\mathrm{CU},\mathrm{CE}\right\}.$$

Table 5 summarizes the existing curves at SNR = 20 dB. Relative to coordinate update, the DP gains are approximately 9.9% and 10.0% for the urban and rural cases, respectively. Relative to cross-entropy, the gains are approximately 20.6% and 17.4%. These are distinct reference comparisons, rather than one common maximum percentage. The array size, channel cases, user count, and processing settings are those specified above; the search-budget interpretation is given in Section 5.3. The DP ordering and partial-sum reuse limit local search work, while the benchmarks evaluate system objectives over their candidate sets. The observed gains support the reported operating points, without establishing superiority at every search budget, an execution-time speedup, or global optimality.

**Figure 10:**
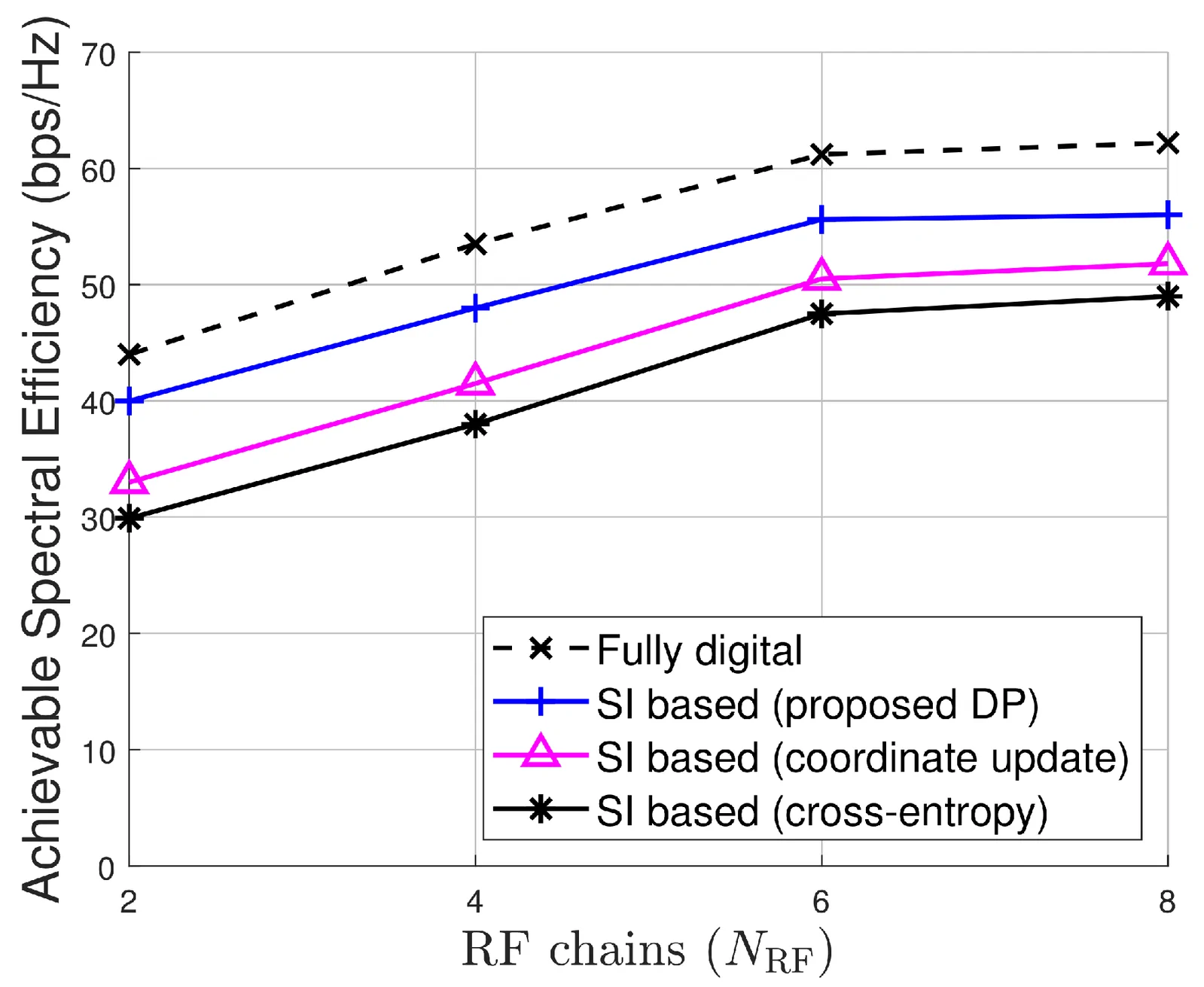
Achievable sum rate versus $N_{RF}$ with $K=N_{RF}$, varied together, at SNR = 20 dB.

With the proposed DP based approach, the SI based architecture achieves a sum-rate about 70% to 80% of that of the PS based architecture, which is an acceptable result considering its relatively low power consumption. To further demonstrate the performance of the SI based architecture, and to provide a like-for-like comparison with the existing methods under identical experimental conditions, the achievable sum-rate is tested under different numbers of RF chains, and the simulation results are shown in Figure 10. Four schemes are included in this comparison: the fully digital ZF precoder, the proposed DP based scheme, the coordinate update scheme [23] and the cross-entropy scheme [22]. The latter three all operate on the same SI architecture described in Section 4, and every scheme is evaluated under exactly the same experimental settings, namely the same UPA with $N_{BS}=16\times 8=128$ antennas, the same inter-element spacing d=λ/2, the same channel realizations generated with the path and angle statistics listed at the beginning of this section, an identical SNR of 20 dB, and results averaged over the same 3000 tests. At each point, all compared schemes serve the same $K=N_{RF}$ users, with the user count varied together with the RF-chain count.

Figure 10 shows that the achievable sum rate grows as the user and RF-chain counts increase together. The proposed DP-based scheme outperforms the coordinate-update and cross-entropy benchmarks over the tested $K=N_{RF}$ configurations, consistently with Figure 8 and Figure 9. This comparison supports the reported performance within that range; it does not establish the effect of additional RF chains at a fixed user count or performance for $K<N_{RF}$. The fully digital ZF precoder is included as the reference without the SI analog constraints.

The observed SI–PS rate difference reflects their different analog constraints, the finite array, and the associated effective-channel and digital-normalization effects. An asymptotic analog-gain ratio cannot directly predict this multi-user sum-rate difference or a fixed loss in dB. The proposed DP-based approach also remains suboptimal in general because it does not examine all feasible sign sequences. The numerical comparisons do not quantify the gap to the globally best analog SI configuration under the prescribed digital-processing rules, and the local example in Section 4.4 cannot determine that sum-rate gap. The comparisons in Figure 10, therefore, describe achieved performance relative to the stated benchmarks, without predicting the benefit of exhaustive search.

### 6.2. Performance of the Antenna Elimination Scheme

The proposed DP-based antenna-deactivation scheme is evaluated using $\xi =R/P_{all}$, with the resulting achievable sum rate from (6) and total consumed power from (7). The numerator includes the effects of the SI analog configuration, digital precoder, and transmit-power normalization; the denominator includes the baseband, RF-chain, transmit, active-switch, and inverter terms. The active-antenna count and retained analog gain guide the design, while the reported EE uses the rate-to-power ratio. The component powers in (7) are set to: $P_{RF}=300$ mW, $P_{BB}=200$ mW, $P_{SW}=5$ mW, $P_{IN}=5$ mW, total transmission power ρ=30 mW under the equal-stream-power convention in Section 3, which is the same as in [22,23]. The power consumption of one PS is set as $P_{PS}=40$ mW for comparison, which is a practical value of a 4-bit PS applied in [7]. We first assume that all the antennas are active, and compare the EE of the SI based architecture with the fully digital and PS based architecture.

**Figure 11:**
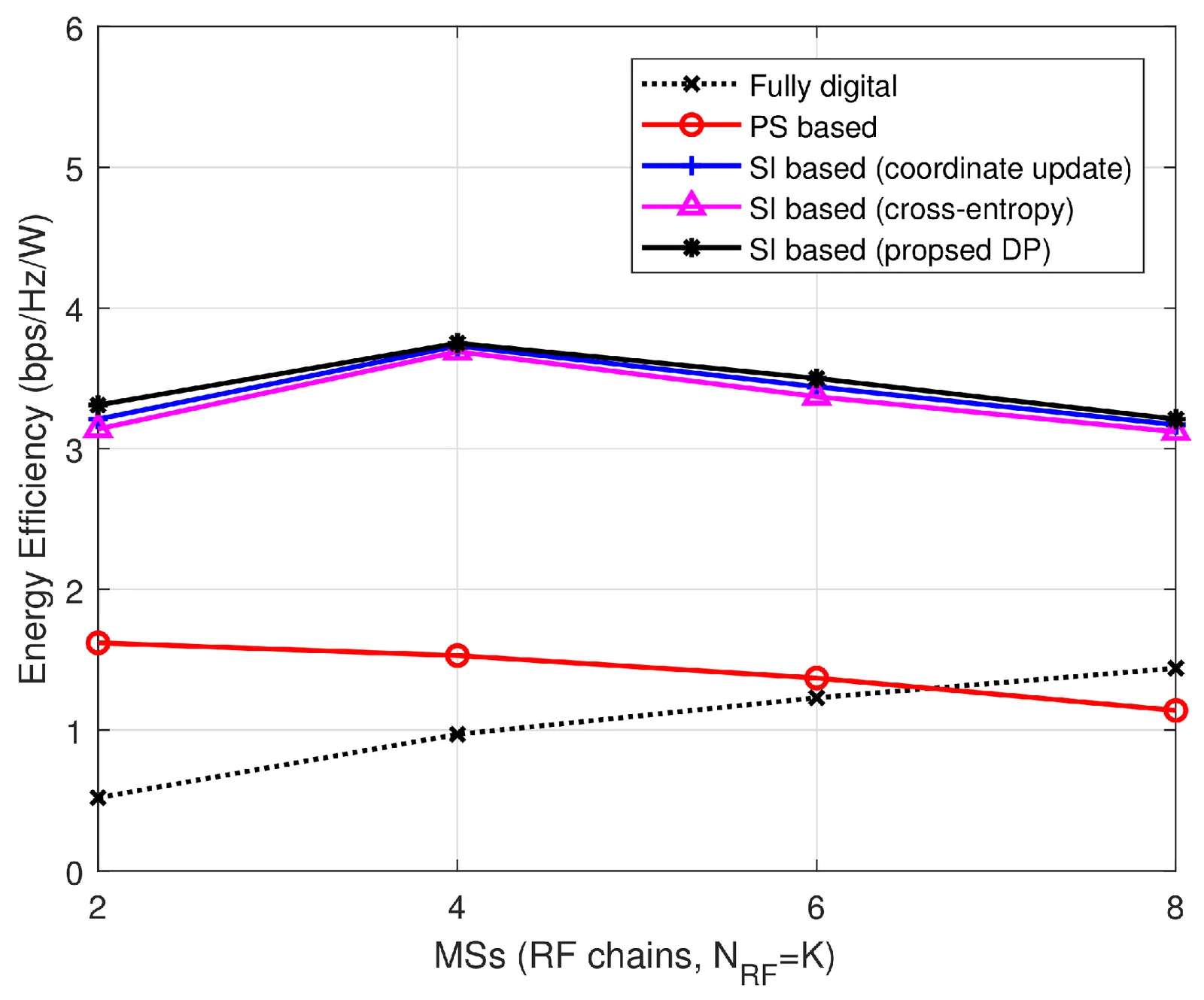
EE versus MSs ($N_{RF}=K$, SNR = 10 dB), with all antennas active.

Figure 11 shows the EE comparison among different architectures and precoding schemes versus a varying number of MSs under SNR=10dB, assuming that all the antennas are active. In the simulations, the number of MSs *K* varies and we assume that the number of RF chains are the same as the number of MSs, as in the benchmark [22]. The resulting EE trends reflect simultaneous changes in served users and RF chains. Under these settings, the SI based architecture exhibits a very high EE compared to the fully digital or conventional architecture using PSs. For the SI architecture, the proposed DP achieves EE close to, or slightly above, the tested benchmarks under these settings. The EE of the fully digital precoder increases when the number of MSs increases, since more MSs improves its overall SE. This is contrast to the PS based architecture, where one more RF chain resulting in $N_{BS}$ more PSs, which is of very high incrementation in power consumption and deteriorates the overall EE. The SI based architecture uses energy efficient switches and inverters, enjoying a relatively high EE in general. However, for the SI based architecture, the EE is decreasing when the number of MSs is becoming larger, which indicates that when the number of MSs is already large enough (K=4 as in the simulation), the SE improvement brought by including one more MS, cannot compensate for the power consumption of one extra RF chain. It is important to note that, due to the high power consumption of RF chains compared to passive analog components, practical millimeter-wave systems typically employ a limited number of RF chains. As the RF chain count increases, the RF front-end power consumption dominates the total system power, thereby reducing the achievable energy efficiency gain.

We then evaluate the EE improvement achieved by the proposed DP based, antenna elimination scheme. For comparison the coordinate update [23] and the cross-entropy schemes [22] are again used, where the latter has to be slightly revised since it does not involve the case where one antenna can be switched off. Therefore, we extend the cross-entropy candidate alphabet for the controllable entries within the prescribed subarrays from {−1,+1} to {−1,0,+1}, while keeping all structural zeros outside those subarrays fixed. This adds antenna deactivation as a selectable state. During the iteration, the candidates with relatively high EE while meeting the QoS constraint are kept for the next generation. The fully digital precoder and the hybrid architecture using PSs, with all antennas active, are also employed to illustrate the EE of the SI based architecture.

**Figure 12:**
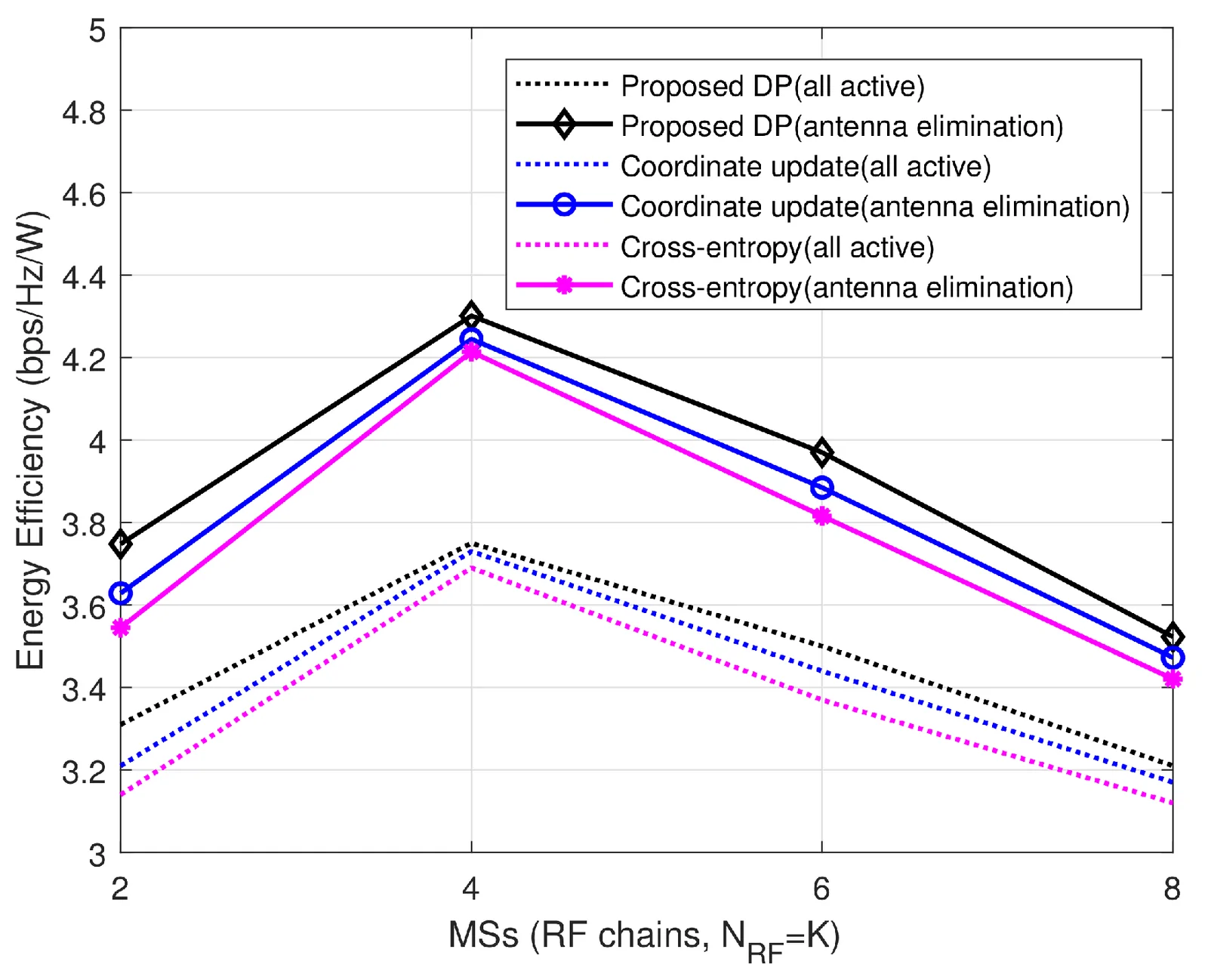
EE versus MSs ($N_{RF}=K$, SNR = 10 dB).

Figure 12 compares EE with and without antenna deactivation. Relative to the corresponding all-active proposed-DP configuration, antenna deactivation gives a reported EE improvement of approximately 15% in the investigated settings. This percentage is not the DP-versus-CU or DP-versus-CE improvement. The 90% QoS setting refers to the retained analog beamforming gain relative to the all-active reference, and does not establish a 10% sum-rate loss. The observed EE improvement reflects the combined change in the actual rate and total power. The proposed scheme outperforms the two tested benchmarks in these cases, supporting the usefulness of contribution-based deactivation under the stated channel and power settings. These comparisons do not establish that the configuration with the fewest active antennas maximizes EE. The RF-chain-dependent trends must also account for the fixed RF-chain power contribution in (7). The computation–performance comparison must likewise include the additional contribution ranking, state updates, and feasibility checks of deactivation, as accounted for in Section 5.3, rather than combining its EE benefit with the cost of all-active construction alone.

### 6.3. Behavior of the Antenna Elimination Scheme

The deactivation order uses beamforming-amplitude contribution estimates. We, therefore, investigate how the gain threshold affects the number of deactivated antennas $N_{BS}^{e}$ and the resulting EE. Relaxing the gain threshold permits more candidate deactivations, but their rate and power changes jointly determine whether EE improves; increased throughput is not implied by a relaxed constraint.

**Figure 13:**
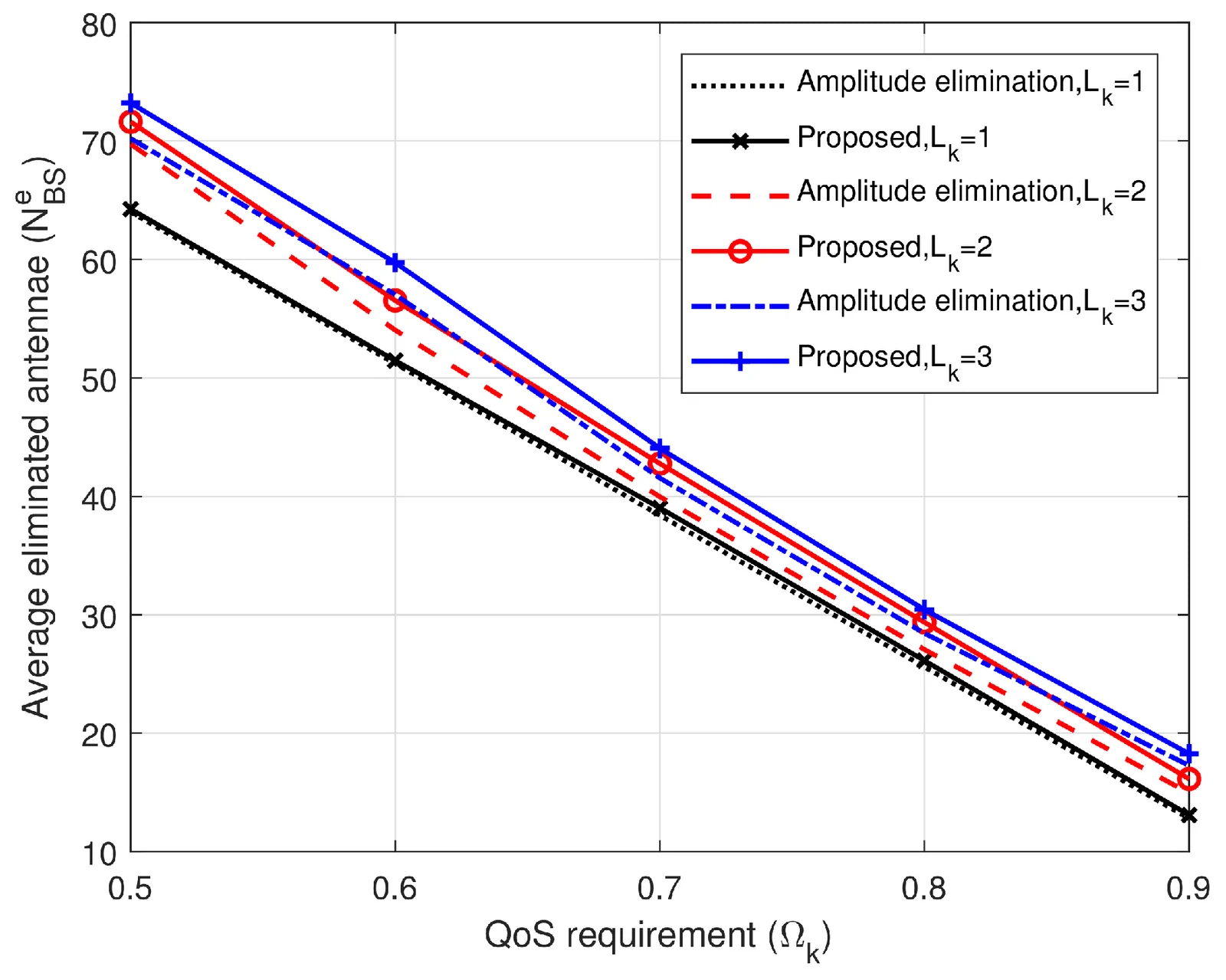
Eliminated antennas $N_{BS}^{e}$ versus QoS requirement $\Omega_{k}$ (SNR = 10 dB).

Figure 13 reports $N_{BS}^{e}$ as the gain threshold varies for $L_{k}=1,2,3$ propagation paths. The amplitude-elimination benchmark ranks antennas only by their coefficient magnitudes, whereas the proposed scheme uses their contributions to the retained partial sums. With multiple paths, the proposed scheme deactivates more antennas at the same gain threshold in the reported cases. This indicates more effective antenna-count reduction under that constraint, rather than a general ordering of EE. For the single-path case with equal coefficient magnitudes, the two strategies give similar deactivation counts. These results support the simpler amplitude-based strategy as an option when computational cost is prioritized, while actual EE must still be evaluated from rate and total power.

**Figure 14:**
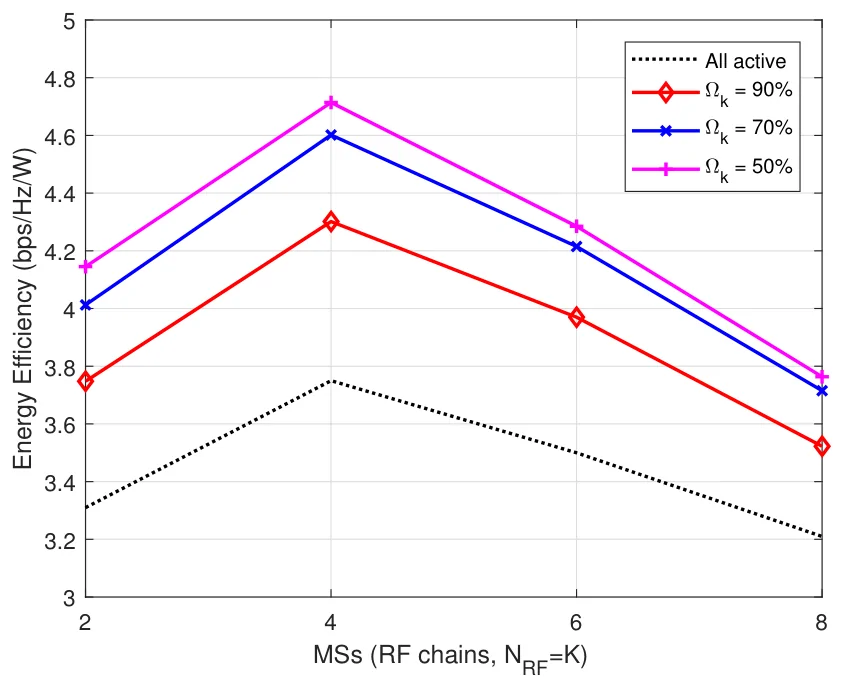
EE versus MSs for different $\Omega_{k}$, with $K=N_{RF}$ varied together (SNR = 10 dB).

Figure 14 shows the resulting EE for different gain thresholds. In the reported settings, relaxing the retained-gain level from 90% toward 50% gives diminishing EE benefits. This illustrates why deactivating more antennas cannot be assumed to improve EE without limit: subsequent removals can cause a relative rate loss that offsets the relative power saving. When RF-chain power dominates $P_{all}$, removing a switch or inverter produces a smaller fractional reduction in total power, so the EE-improvement condition becomes more restrictive. These observations are consistent with the rate–power trade-off analyzed after (24); they are scenario-specific evidence of EE improvement, not a bound on the gap to the global EE optimum.

### 6.4. Robustness Against Imperfect CSI

**Figure 15:**
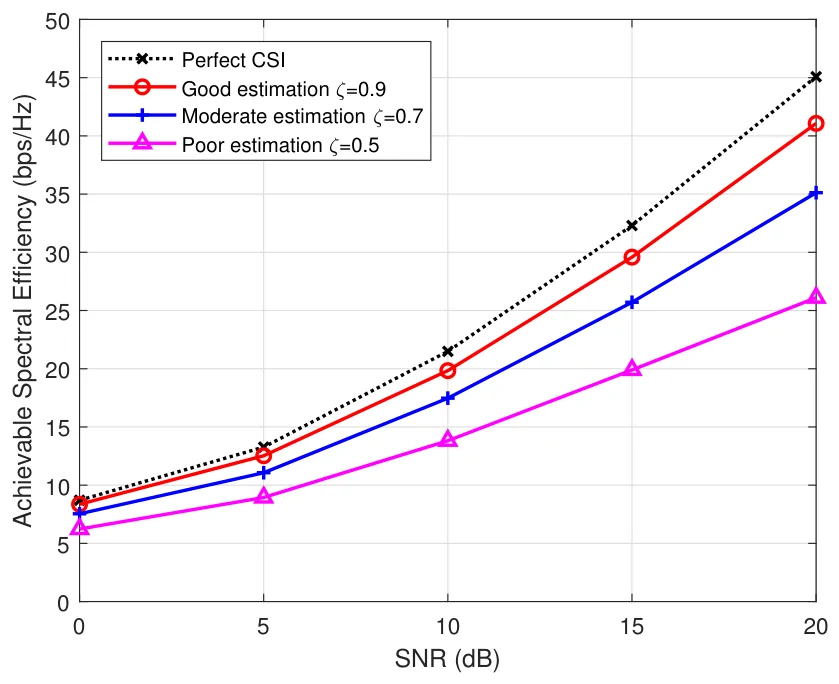
Impact of imperfect CSI (all antennas are active).

Finally, we evaluate the impact of imperfect CSI on the proposed DP based approach, since it depends heavily on the estimated entry-wise channel vector $h_{k}$, which is not very easily obtained for a massive array in practice. We use the channel-perturbation model of Gao et al. [10] (Equation (22)), written here for the channel vector of each user rather than the full channel matrix:

(30) $${\hat{h}}_{k}=\zeta h_{k}+\sqrt{1-\zeta^{2}}e,$$

where $\zeta \in \left[0,1\right]$ controls the weights of the true channel and the perturbation, and $e\in C^{N_{BS}\times 1}$ is an error vector whose entries follow the i.i.d. ${\left[e\right]}_{i}∼\mathrm{CN}\left(0,1\right)$. The symbol ζ corresponds to the accuracy parameter in the cited model and is distinct from our EE symbol ξ. We retain the path-gain scaling in (3) and use (30) as a prescribed sensitivity model; ζ is not asserted to equal a normalized channel-correlation coefficient under arbitrary channel and error covariances. Figure 15 demonstrates the impact of imperfect CSI for different ζ, where ζ=0.9, 0.7, and 0.5 specify progressively larger perturbation weights. When the perturbation weight becomes sufficiently small, the system performance gradually approaches that of the ideal CSI case. The perfect-CSI results serve as a reference for this sensitivity evaluation, rather than a certified performance bound on every realization of the heuristic algorithm.

At the reported operating points in Figure 15, the sum rate is approximately 90%, 80%, and 60% of the perfect-CSI reference for ζ=0.9, 0.7, and 0.5, respectively. These are observations for the evaluated channel and processing settings, rather than universal robustness guarantees.

CSI errors can perturb the local coefficients and change the preferred SI signs, providing a qualitative connection to the phase-sensitive comparisons in Section 5.2. They also affect the effective-channel estimate used for digital ZF. Consequently, rate loss can arise through residual inter-user interference or altered precoder normalization even when the SI signs remain unchanged. The error entries in (30) are random complex Gaussian variables, not deterministic real perturbations of fixed magnitude. The local angular analysis, therefore, does not establish a universal phase-inversion threshold in ζ or quantify the complete multi-user rate loss.

The simulations assume additive white Gaussian noise and ideal hardware responses. In practical mmWave systems, additional receiver noise, phase errors, and hardware distortion may reduce the achievable rate and, at unchanged total consumed power, EE. These impairments can affect both analog beamforming gain and digital ZF interference suppression, potentially changing the relative performance of the compared schemes. The imperfect-CSI results characterize channel-estimation sensitivity rather than general hardware-impairment robustness.

## 7. Conclusions

In this paper, we have studied SI analog-precoder design within a multi-user hybrid precoding architecture under a frequency-flat narrowband channel model. The proposed DP procedure selects local SI analog weights with low computational complexity, and the associated digital ZF precoder is computed from the resulting effective multi-user channel. EE is evaluated by $R/P_{all}$ throughout this work. To pursue an improved rate–power trade-off, the revised DP approach switches off selected antennas while meeting a predefined analog-gain QoS requirement. The analysis distinguishes exact comparisons within the retained candidate set from global optimization and uses illustrative cases to explain the remaining suboptimality. No universal approximation bound for the proposed algorithm is established. The antenna-count formulation is a QoS-constrained surrogate for reducing circuit power; the analysis specifies the relative rate-loss condition for EE improvement and the additional assumptions required for equivalence or near-equivalence to EE maximization. Simulation results demonstrate performance advantages over the tested benchmarks for the evaluated $K=N_{RF}$ configurations. The system and ZF formulation permit $K\leq N_{RF}$ under the full-row-rank condition, while performance for additional RF chains at a fixed user count is not evaluated here.

The discussion in Section 4.5 identifies a sufficient proportional-channel condition under which the ordering of local analog beamforming gains is consistent across subcarriers. General frequency-selective operation requires a common analog network designed jointly across subcarriers and digital precoders adapted to their individual effective channels. Its sum-rate, QoS, and EE properties require a separate joint analysis. For future work, it will also be of interest to investigate methods for further power consumption reduction of the SI based architecture, for example, deactivating some inverters or switches at the expense of relatively small performance degradation.

## Figures and Tables

**Table 1:** Representative hybrid architectures and algorithms compared with the proposed SI method.

Approach	Architecture and Variables	Design Focus/Mechanism	Relation to This Work
PS-based hybrid [4,6,9,20]	Analog phases; digital weights	Sparse, iterative, or channel-hardening design	Phase-shifter constraints; SI uses sign/off states
Two-layer PSs [18]	Low-resolution phases; layered codebooks	Adaptive-weighted CE or greedy search	Two-layer PS/codebook design
Switch-only [7]	Binary on/off paths	Hardware and rate–power trade-offs	SI additionally permits sign −1
SI CE/CU [22,23]	Discrete SI states	Sampling or coordinate-wise search	Same SI hardware; different candidate evaluation and reuse
Dynamic subarrays [11,19,21]	RF connections; analog phases	Adaptive subarray mapping	Our RF-chain mappings stay fixed
Wideband hybrid [15,16]	Shared analog; per-subcarrier digital weights	Frequency-domain joint design	Here: frequency-flat model; conditional extension
Antenna selection/combining [25,26,28,29]	Active antenna/RF subsets	Greedy, convex, decremental, or message-passing selection	Here: transmitter SI activation with fixed RF chains
Subspace-based hybrid [21]	PS phases; digital weights; optional partitioning	Subspace RF design; reduced digital optimization	PS constraints; adaptive antenna mapping supported
Learning-assisted analog [30]	Trained beamforming policy	Reinforcement learning	Training requirements differ from direct DP updates
Proposed method	Fixed SI subarrays; signs and activation	Partial sums, ordering, and gain-constrained deactivation	Local comparisons; no global optimality guarantee

**Table 2:** Search mechanisms and the associated computational trade-offs.

Method	Candidate Evaluation and Reuse	Search Budget and Limitation
Revised DP	Two-sign or four-combination local amplitude comparison; reuse of complex partial sums	Ordered sequential processing and affected-state updates; discarded histories can exclude better continuations
Cross-entropy	Complete analog candidates with effective-channel, digital-precoder, and objective evaluation	Candidate count *S* and iteration count *I*; sampling and distribution updates depend on this budget
Coordinate update	Alternative antenna states with the remaining states fixed; system-objective evaluation	Number of coordinates, tested states, and scans $t_{\max}$; local updates depend on initialization and the scan budget

**Table 3:** Whole-array and antenna-normalized workload measures. All numerical examples use $N_{BS}=128$, $K=N_{RF}=4$, and $n_{i}=32$. Evaluation counts and asymptotic arithmetic orders are identified separately; these are not measured FLOPs or execution times.

Method/Stage	Whole-Array Workload	Per-Installed-Antenna Measure	Unit and Cost Coverage
DP, all-active	At most 496 local amplitude evaluations; sorting $O(\sum_{i}n_{i}\log n_{i})$	At most 3.875 local evaluations; average sorting $O(\log\left(N_{BS}/N_{RF}\right))$	One pass per subarray; digital processing and deactivation costs are additional
DP, with deactivation	Cost model $C_{\mathrm{DP}}$ above, with sign-repair visits *L*, *T* trial checks, and *Q* rate/EE evaluations	$O(\log N_{BS}+L/N_{BS})$ plus $O(\left(TC_{G}+QC_{E}\right)/N_{BS})$	Complete trial reconstruction remains in $C_{G}$, including after a heuristic stop
Cross-entropy	4000 complete candidate evaluations; dominant arithmetic $O(ISN_{BS}K^{2})$	Dominant arithmetic $O(ISK^{2})$	I=20, S=200; each candidate covers the full array; other search overhead is additional
CU, binary	7680 principal coordinate-candidate objective evaluations	60 objective evaluations per antenna	q=2, $t_{\max}=30$; multiply the count by the per-evaluation cost to obtain work
CU, ternary	11,520 principal coordinate-candidate objective evaluations	90 objective evaluations per antenna	q=3, $t_{\max}=30$; includes state 0; each evaluation uses system-level processing

**Table 4:** Reported defaults of the frequency-flat sparse geometric channel simulations. User and RF-chain counts are varied together where indicated; other departures from these defaults are stated in the relevant comparisons.

Parameter	Simplified Urban Setting	Simplified Rural Setting
Propagation paths per user	$L_{k}∼U\left\{1,2,3\right\}$	$L_{k}=1$
Complex path gain	$g_{k,l}∼\mathrm{CN}\left(1,1\right)$	$g_{k,1}∼\mathrm{CN}\left(1,1\right)$
Polar angle from array normal	θ∼U(0,π/2)	θ∼U(0,π/2)
Azimuth angle	ϕ∼U(0,2π)	ϕ∼U(0,2π)
Cluster/ray hierarchy	Not separately modeled	Not separately modeled
Cluster angular spread	Not specified	Not specified
Distance-dependent path loss	Not modeled; no exponent assigned	Not modeled; no exponent assigned
BS array	16×8 UPA, $N_{BS}=128$	16×8 UPA, $N_{BS}=128$
Element spacing	d=λ/2	d=λ/2
Default users/RF chains	$K=N_{RF}=4$	$K=N_{RF}=4$
Default analog-gain threshold	90% of corresponding all-active gain	90% of corresponding all-active gain
Averaging	3000 trials	3000 trials

**Table 5:** Sum-rate comparison at SNR = 20 dB for the all-active 128-antenna SI array with $K=N_{RF}=4$. Rates are in bit/s/Hz. Values are approximate, recovered from the existing plotted curves and rounded; percentage gains use the unrounded plotted values.

Channel Case	DP Rate	CU Rate	CE Rate	Gain vs. CU	Gain vs. CE
Urban	45.02	40.97	37.33	9.9%	20.6%
Rural	50.70	46.10	43.20	10.0%	17.4%

## Data Availability

Data supporting the findings of this study are available from the corresponding authors upon reasonable request.

## Abbreviations

The following abbreviations are used in this manuscript:

**Abbreviation**	**Full term**
BS	Base station
CE	Cross-entropy
CSI	Channel state information
CU	Coordinate update
DAC	Digital-to-analog converter
DoD	Direction of departure
DP	Dynamic programming
DQSM	Double quadrature spatial modulation
EE	Energy efficiency
FLOP	Floating-point operation
LDPC	Low-density parity-check
MIMO	Multiple-input multiple-output
mmWave	Millimeter-wave
MS	Mobile station
OFDM	Orthogonal frequency-division multiplexing
PS	Phase shifter
QoS	Quality of service
RF	Radio frequency
RIS	Reconfigurable intelligent surface
SE	Spectral efficiency
SI	Switch-inverter
SINR	Signal-to-interference-plus-noise ratio
SNR	Signal-to-noise ratio
TDD	Time-division duplex
ULA	Uniform linear array
UPA	Uniform planar array
ZF	Zero-forcing
